# Molecular connectivity–driven tuning of aggregation-induced emission and mechanoresponsive properties in fused TPE–carbazole materials

**DOI:** 10.1039/d6tc01521b

**Published:** 2026-08-21

**Authors:** Dylan Wilkinson, Madeleine McRoberts, Abdul-Wasir Shaka, Sarah K. Dugmore, Claire Wilson, Pablo Docampo, Malcolm Kadodwala, Graeme Cooke

**Affiliations:** a School of Chemistry, University of Glasgow, University Place Glasgow G12 8QQ UK greame.cooke@glasgow.ac.uk; b Basque Center for Materials, Applications and Nanostructures (BCMaterials) 48940 Leioa Spain

## Abstract

Precise control of solid-state fluorescence through molecular design is central to the development of advanced functional materials for sensing, imaging, and optical information storage. In particular, aggregation-induced emission (AIE) and mechanochromic luminogens have emerged as promising platforms for stimuli-responsive photonic applications. Herein, four constitutional isomers of carbonyl-bridged tetraphenylethylene (TPE)–carbazole derivatives were synthesised to systematically investigate how molecular connectivity governs their optoelectronic, aggregation, and mechanoresponsive properties. Distinct structure–property relationships were established, demonstrating that subtle variations in connectivity produce significant differences in electronic structure, molecular packing, aggregation-induced emission, and mechanochromic behaviour. Compounds 1, 3, and 4 exhibit AIE characteristics, whereas compound 2 displays aggregation-caused quenching-like behaviour arising from differences in accessible conformational and solid-state packing states. Among the four isomers, compounds 1 and 2 exhibit mechanochromism, with compound 1 showing highly reversible fluorescence switching, while compound 2 undergoes an irreversible pressure-induced transition associated with a metastable amorphous phase. Fluorescence microscopy revealed an exceptionally low pressure-sensing threshold of 0.2 MPa for compound 1 deposited on paper substrates, enabling sensitive detection of gentle mechanical stimuli and reversible invisible-ink write–erase cycles. These findings establish constitutional isomerism as an effective strategy for controlling aggregation, solid-state organisation, pressure sensitivity, and mechanochromic reversibility, providing molecular design principles for next-generation low-force pressure sensors and stimulus-responsive organic materials.

## Abstract 2

Isomeric carbonyl-bridged TPEs exhibit structure-dependent mechanochromism, enabling reversible low-pressure sensing, invisible ink, and phase-switchable solid-state emission.

## Graphical abstract

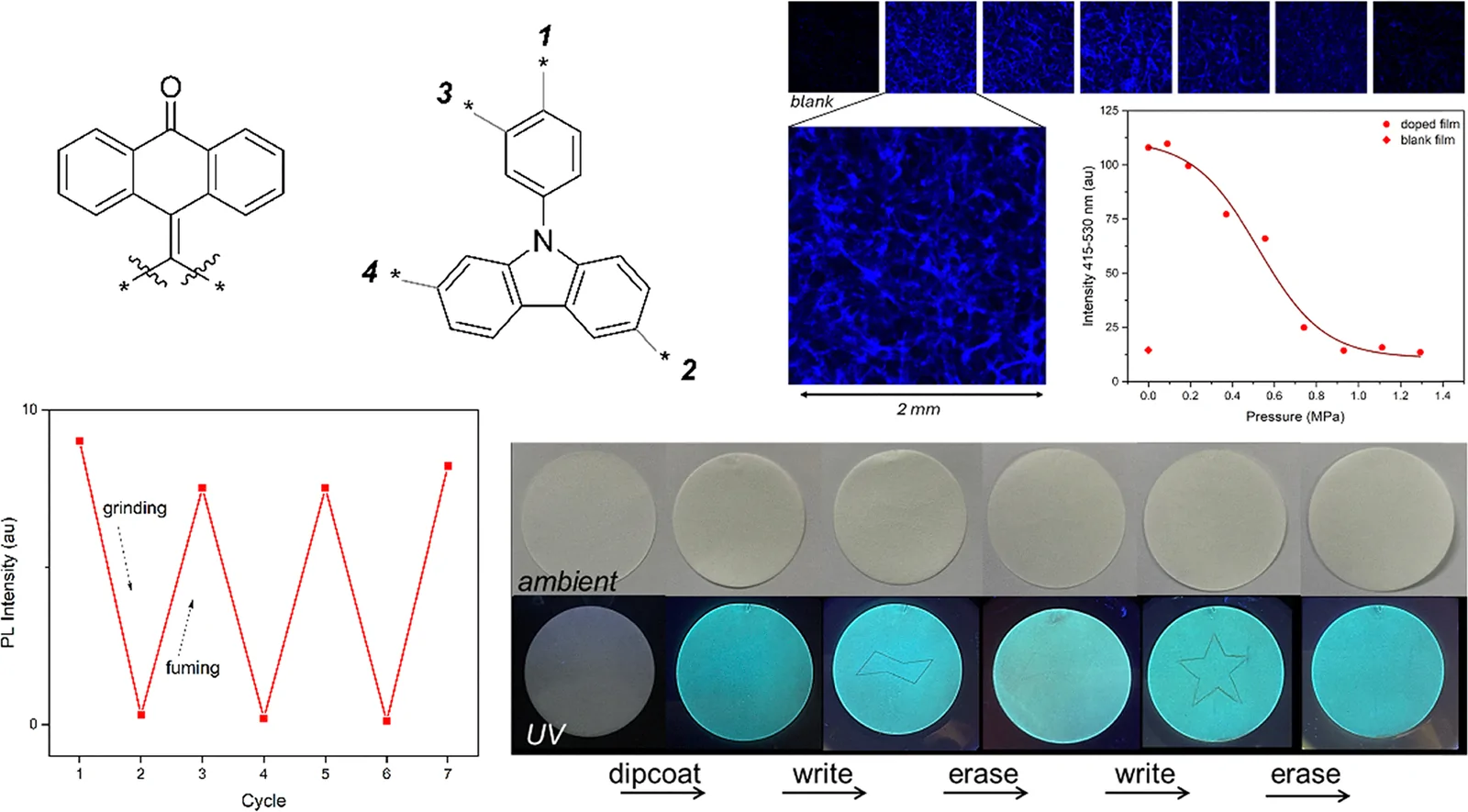

## 1. Introduction

Stimuli-responsive luminescent materials have emerged as a dynamic class of functional systems with broad implications for sensing, security, data storage, and smart optoelectronic devices.^1–7^ Among these, fluorescent mechanochromic materials, chromophores capable of altering their emissive properties in response to external mechanical forces such as grinding, stretching, or pressing, have attracted particular attention.^8–12^ Their mechanically induced colour changes often arise from alterations in molecular packing, conformational rearrangements, or phase transitions within the solid state.^13–15^ This sensitivity to subtle structural variations enables mechanochromic fluorophores to act as powerful reporters of mechanical stress and microenvironmental change, positioning them as promising candidates for pressure sensors, rewritable media, and structural health monitoring technologies.^16–21^ In particular, materials that exhibit luminescence changes under very small applied pressures are highly desirable for low-force mechanical sensing, flexible and wearable devices, and reusable invisible inks, where rapid, reversible responses to gentle mechanical stimuli are essential.

Alongside advances in mechanochromism, the discovery of aggregation-induced emission (AIE) has reshaped luminescent material design.^22–24^ Unlike conventional fluorophores prone to aggregation-caused quenching, AIE-active molecules become more emissive when aggregated due to restricted intramolecular motions. This property has enabled materials with high solid-state quantum yields, good processability, and tuneable emission, leading to broad use of AIE luminogens in bioimaging, sensing, photodynamic therapy, and optoelectronic devices.^25–29^ The intrinsically nonplanar conformations common to many AIE-active molecules are well suited to enabling luminescence that responds to mechanical stimuli. Consequently, creating luminogens that combine both AIE and mechanofluorochromic properties is crucial for broadening their functional scope and expanding their potential applications in this field.^30–32^ Understanding the molecular design strategies, structure–property relationships, and mechanistic underpinnings of these materials is essential for expanding their functionality and unlocking new applications in smart, responsive photonic systems.

Tetraphenylethylene (TPE) based materials have been widely studied as AIE-gens and are reported to have mechanochromic properties.^33–35^ These materials have recently been utilised in anti-counterfeiting applications, pressure detection and analyte sensing.^36,37^ Y. Cai *et al.* reported sulphur bridged fused TPE materials as deep blue emitting AIE-gens with promising OLED performance.^38^

Despite these advances, most studies on TPE-based AIE luminogens and mechanochromic materials have focused on developing new molecular architectures or improving luminescence performance. In comparison, systematic investigations into how constitutional isomeric connectivity influences pressure-responsive behaviour remain limited, particularly within homologous TPE–carbazole systems. Because changes in molecular connectivity can modify molecular conformation, intermolecular interactions, crystal packing, and electronic coupling, they are expected to strongly influence key performance metrics, including pressure-sensing thresholds, reversibility, and mechanochromic response. Establishing these structure–property relationships is therefore important for the rational design of highly sensitive and reversible pressure-responsive organic materials.

Herein, we report the design and synthesis of a series of donor–acceptor fused tetraphenylethene (TPE) derivatives incorporating a carbonyl bridge between two TPE rotors (Fig. 1). Integration of *N*-phenylcarbazole donor units with an anthraquinone-based acceptor core afforded four isomeric donor–acceptor (push–pull) molecules with intriguing optoelectronic properties. The molecular design combines strong electronic communication with freely rotating subunits, thereby promoting aggregation-induced emission (AIE) and mechanochromic behaviour. Carbazole derivatives are widely employed building blocks in organic electronics due to their low cost, synthetic versatility, excellent optoelectronic characteristics, and high chemical and thermal stability.^39^ A series of four constitutional isomeric carbazole-based fused TPE compounds (1–4) was synthesised, enabling a systematic investigation of how isomeric connectivity governs the optoelectronic properties, aggregation-induced emission, pressure-sensing behaviour, and mechanochromic reversibility of this homologous molecular family. Comprehensive optoelectronic, electrochemical, crystallographic, and computational studies demonstrate that the optoelectronic, aggregation, and mechanoresponsive properties of fused TPE materials can be finely tuned through deliberate structural design. In particular, this work elucidates clear structure–property relationships, highlighting how variations in the connectivity between the *N*-phenylcarbazole units and the central core govern electronic structure, aggregation behaviour, crystal packing, pressure-sensing thresholds, mechanochromic reversibility, and solid-state optical performance.

**Fig. 1:**
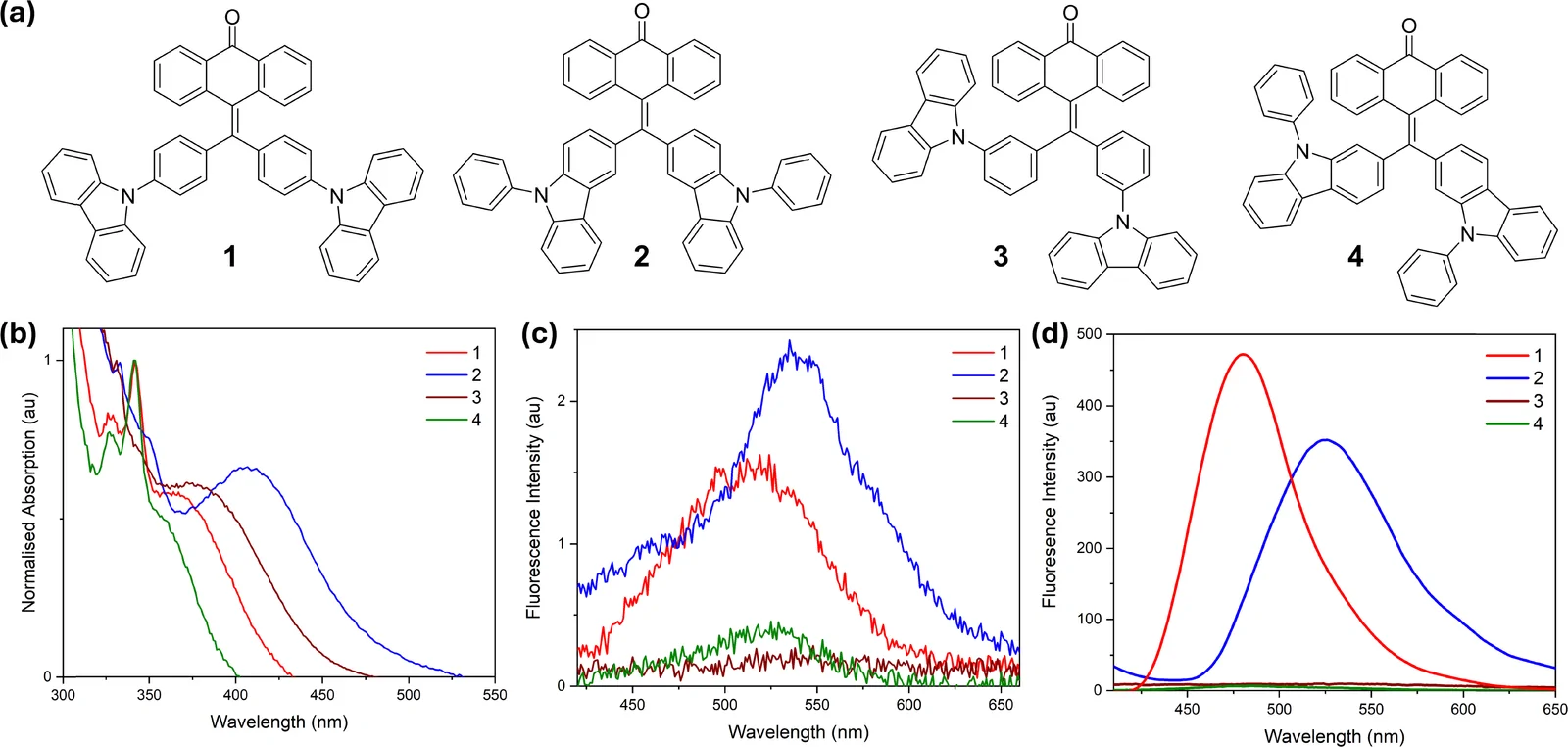
(a) Structure of isomeric compounds 1–4. (b) Normalised absorption spectra (1 × 10^−5^ mol L^−1^ chloroform solution), (c) emission spectra (1 × 10^−3^ mol L^−1^ chloroform solution, excited at 350 nm), and (d) solid-state emission spectra of compounds 1–4.

## 2. Results & discussion

### 2.1. Synthesis

For all targets, the proposed synthetic route begins with a modified Corey–Fuchs reaction with anthraquinone as described in the literature to produce subsequent dibromo compound 10-dibromomethylene-9-anthrone (DBMA) (Scheme 1).^40^ This was followed by a double Suzuki reaction to attach *N*-phenylcarbazole groups and assemble the TPE core yielding targets 1–4. All boronic acids were commercially available. Standard Suzuki conditions were used for each reaction, and products were formed in moderate to excellent yields.

**Scheme 1:**
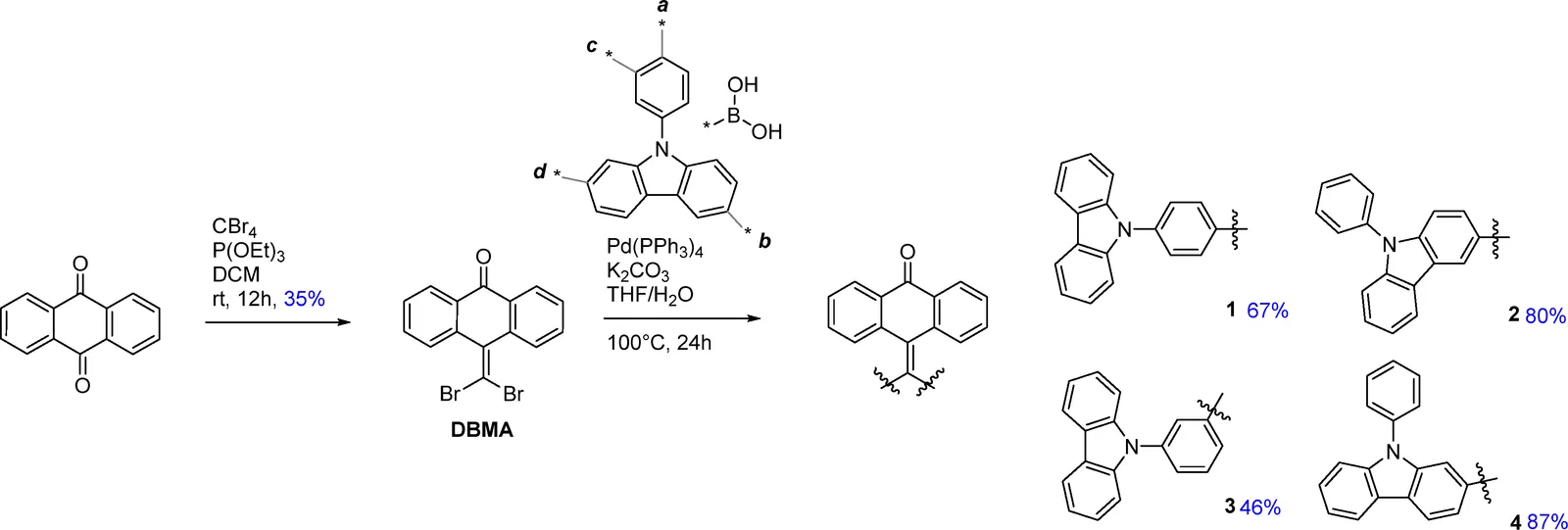
Synthesis of fused-TPE target materials 1–4 starting from anthraquinone. For Suzuki coupling step, asterisk (*) indicates position of boronic acid group on *N*-phenylcarbazole reagent. Boronic acids a, b, c and d correspond to products 1, 2, 3 and 4 respectively.

### 2.2. Characterisation

The absorption spectrum for each compound was measured in chloroform solution. Each compound exhibited two main distinct absorption events; a peak at ∼340 nm and then a low energy absorption peak between 360–500 nm. The peak at 340 nm can be ascribed to π → π* transition within the conjugated core of the molecule. The low energy absorption peak varies in intensity and wavelength and can be attributed to intramolecular charge transfer between donor and acceptor moieties. Compound 1 has an absorption maximum at 366 nm whereas associated isomeric compounds, 2, 3 and 4 have different absorption properties. Compounds 2 and 3 exhibit intense peaks which are red shifted compared with 1 to 407 nm and 379 nm, respectively. The transition for 4 is blue shifted to 375 nm and is partially obscured by peak at 340 nm. It is speculated that the conjugated backbone of each isomer has an influence on the charge transfer character of this peak. In 1, 2 and 3, electron density is conjugated from donor to acceptor, whereas in 4, due to the *meta* position of the carbazole group with respect to the central phenyl group, leading to inefficient conjugation between donor and acceptor. This disconnection leads to blue shifting and a reduced intensity CT band.

The solution fluorescence spectra were recorded for all four compounds, revealing low intensity emissions (Fig. 1). All compounds have a relatively large Stokes shift and display a single emission peak between 500–575 nm. Compound 1 has maximum at 515 nm, red shifted by 149 nm from absorption (7905 cm^−1^ Stokes shift), while compounds 2 and 4 have emission peaks at 540 nm and 520 nm, with Stokes shift of 133 nm (6052 cm^−1^) and 145 nm (7154 cm^−1^) respectively. By design these molecules were anticipated to have weak emission in solution due to rotational freedom. The large Stokes shifts arise from significant structural reorganisation in the excited state due to molecular motion. The optoelectronic properties of the solid state and aggregates were investigated. The fluorescence intensity varied significantly, with 3 and 4 being almost completely non-emissive in the solid state, while 1 and 2 gave high intensity emission in the region between 480 and 525 nm, respectively. In the solid state all compounds emit at a lower wavelength than in solution, which is attributed to suppression of freedom of molecular motion, therefore the Stokes shift is attributed to vibrational relaxation only.

Cyclic voltammetry (CV) was carried out to estimate the ionisation potentials and electron affinities of the compounds (Fig. 2). All compounds exhibit oxidation processes within the potential range 0.0–1.5 V *versus* Fc/Fc^+^ and reduction processes for all compounds occur near the cathodic limit of the solvent window, with quasi-reversible reduction waves observed between −1.2 and −2.0 V *versus* Fc/Fc^+^. The ionisation potential and electron affinity were estimated from the first oxidation and reduction potentials using the Fc/Fc^+^ redox couple as an internal reference and assuming an energy of −4.8 eV relative to vacuum. The ionisation potential and electron affinity were calculated according to IP = −(*E*_ox_ + 4.8) and EA = −(*E*_red_ + 4.8), respectively. Compounds 1–4 therefore exhibited estimated IP energies of −5.4, −5.1, −5.4 and −5.3 eV, respectively, with corresponding EA energies of −3.2, −3.3, −3.3 and −3.2 eV. The resulting electrochemical energy gaps were 2.2, 1.8, 2.1 and 2.1 eV for compounds 1–4, respectively. Compound 2 has the smallest bandgap, in agreement with the optical absorption.

**Fig. 2:**
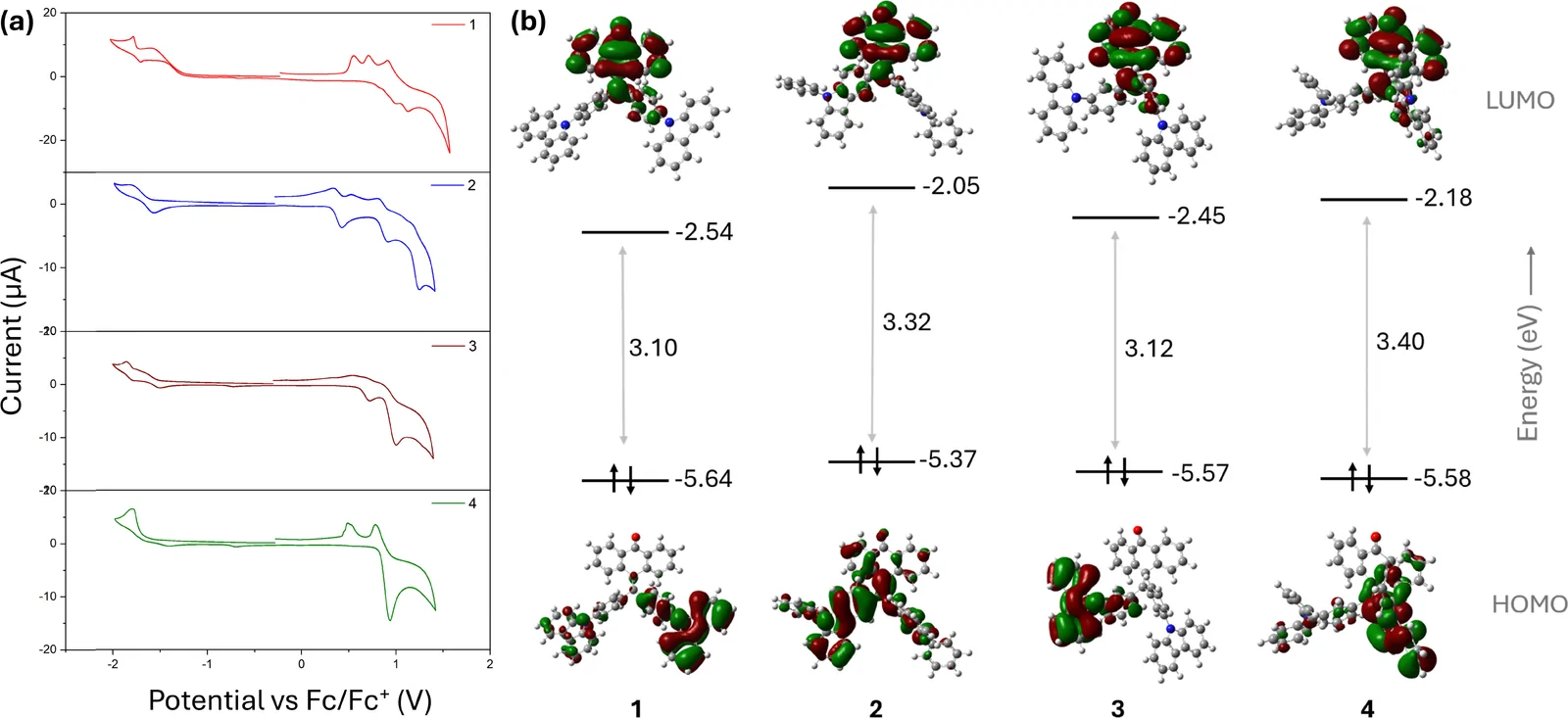
(a) Cyclic voltammetry of 1–4 showing reduction and oxidation peaks *versus* Fc/Fc^+^; (b) frontier molecular orbitals and resulting band gap of 1–4 calculated using Gaussian 16 DFT B3LYP functional with the 6-311G(d,p) basis set.

Density functional theory (DFT) calculations were performed to examine the frontier molecular orbital (FMO) energies of all compounds (Fig. 2). The isomeric compounds display a broad range of FMO energy levels and associated band gaps, underscoring the significant influence of molecular connectivity on the electronic properties. The computed HOMO energies span −5.64 to −5.37 eV, while the LUMO energies range from −2.54 to −2.05 eV. For all compounds, the LUMO is predominantly localised on the bridging acceptor moiety and the central core. In contrast, the HOMO is primarily distributed over the donor carbazole units. Notably, compounds 2 and 4 display relatively diffuse HOMO distributions, with significant electron density on the central core. Compounds 1 and 3 presents the clearest divergence of HOMO and LUMO electron density distribution, potentially because of the contorted structure, enforced by the orientation of the *N*-phenylcarbazole unit relative to the core, which prohibits efficient orbital overlap. Although the optical and electrochemical characterisation suggests that compound 2 possesses the smallest bandgap, it should be noted that the DFT calculations were performed only on the lowest-energy conformer. Owing to the conformational flexibility of these compounds, multiple conformers are accessible depending on the environment and experimental conditions. When conformers 1B and 2B are also considered, substantially smaller bandgaps are calculated (Fig. S15). These lower-energy conformers therefore provide a plausible explanation for the lower-energy absorption features observed experimentally, highlighting the importance of considering the full conformational landscape when interpreting the electronic structure of these systems. Further detailed discussion of the polymorphic behaviour of these compounds is included in Sections 2.3 and 2.4.

Crystals of all compounds were grown using vapor diffusion method and single crystals of compounds 2, 3 and 4 were successfully isolated and analysed by using single crystal X-ray diffractometry and the structures were solved and refined (Fig. 3). Unfortunately, compound 1 gave small fibril type crystals unsuitable for XRD analysis. The single crystal of compound 2 revealed interlocked π–π stacking of each molecule, with molecules stacked in a head-to-head orientation. π–π stacking distances of 3.0 and 2.9 Å were recorded between headgroup protons of adjacent molecules, and headgroup protons to nitrogen of *N*-heterocarbazole fragment respectively. In compound 3, the molecules are far more contorted and heavily intertwined in a complex three-dimensional zig-zag orientation. A π–π stacking distance of 3.1 Å was recorded between carbonyl groups and adjacent aryl protons, and a distance of 2.9 Å was recorded between adjacent *N*-heterocarbazole fragments. Compound 4 exhibits a columnar like packing conformation where the carbazole groups of adjacent molecules interlock through π–π stacking at distance of 2.5 Å. An intermolecular distance of 3.5 Å was recorded between carbonyl oxygen and aromatic protons. In general, it is clear that in each case, the molecular crystal packing is drastically affected by molecular connectivity, and therefore provides further evidence for discrete optoelectronic and mechanochromic properties of the isomers.

**Fig. 3:**
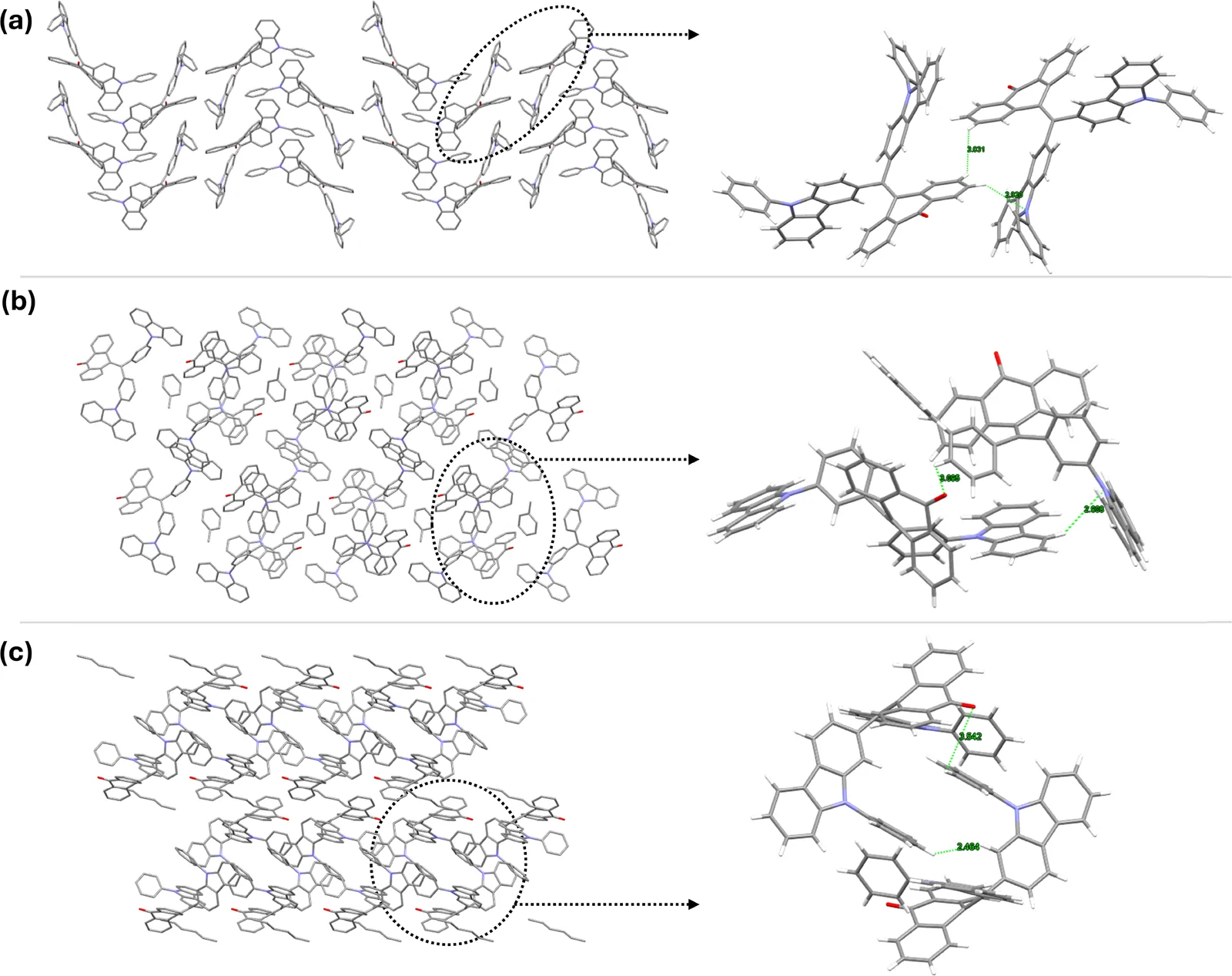
Single crystal XRD data of (a) 2, (b) 3, and (c) 4 showing 2 × 2 × 2 unit cell packing viewed down the ac projection plane with hydrogen atoms removed for clarity. A magnified section of the crystal structure reveals intermolecular packing distances given in angstrom (Å).

### 2.3. Aggregation induced emission (AIE)

The aggregation properties of each compound were investigated using standard aggregation-induced emission (AIE) co-solvent experiments (Fig. 4).^23^ Tetrahydrofuran (THF) was selected as a good solvent, while water served as a poor solvent. Solutions (5 × 10^−4^ mol L^−1^) containing varying water fractions (0–90 vol%) were prepared, and their fluorescence spectra were recorded. For AIE-active molecules, increasing the proportion of the poor solvent reduces solubility, promoting aggregation. The resulting restriction of intramolecular motions (RIM) suppresses non-radiative decay pathways and enhances fluorescence emission.

**Fig. 4:**
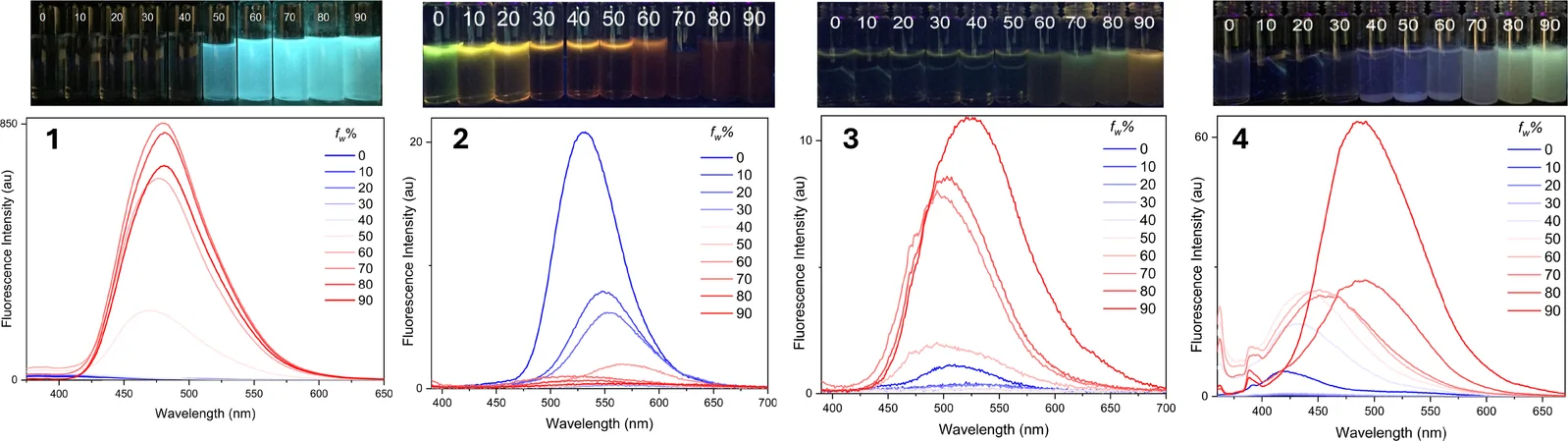
Aggregation induced emission co-solvent experiments for compounds 1–4. Fluorescence intensity measured of THF/water solutions prepared at 1 × 10^−5^ with ratio of water (*f*_w_%) increasing from 0 to 90%. Images above of solutions captured under 365 nm UV lamp illumination.

Compound 1 exhibited classical AIE behaviour. From 0 to 40 vol% water, the molecule remained molecularly dissolved and fluorescence was almost completely quenched. Above 50 vol% water, aggregation occurred and the emission intensity increased markedly, producing blue fluorescence with an emission maximum at approximately 480 nm. Compound 3 displayed a similar, although less pronounced, response. Fluorescence emerged above 50 vol% water with emission centred near 500 nm. At 90 vol% water, a further red shift to approximately 530 nm was observed, which is attributed to precipitation of the material into a bulk solid phase rather than dispersed aggregates. Compound 4 also exhibited weak AIE behaviour, with fluorescence appearing above 40 vol% water and gradually red-shifting from approximately 430 to 500 nm as the water fraction increased.

The differing aggregation behaviour of compounds 1, 3, and 4 demonstrates that the AIE response is highly sensitive to constitutional isomerism. Although the molecular composition of the four compounds differs only in the connectivity of the *N*-phenylcarbazole donor units to the fused TPE framework, single-crystal X-ray diffraction reveals that each isomer adopts a distinct three-dimensional molecular conformation and crystal packing arrangement (Fig. 3). These structural differences influence both the rotational freedom of the aromatic subunits in solution and the extent to which these motions become restricted upon aggregation. Consequently, the efficiency of the RIM mechanism differs between the constitutional isomers, leading to substantial variations in fluorescence enhancement in the aggregated state. These results highlight that molecular connectivity provides an effective means of tuning AIE behaviour through its influence on molecular geometry and solid-state organisation.

Interestingly, compound 2 did not exhibit conventional AIE behaviour. Instead, its fluorescence intensity decreased progressively with increasing water fraction, displaying apparent aggregation-caused quenching (ACQ)-like behaviour. At first glance, this appears inconsistent with the intense fluorescence observed for the pristine crystalline solid (Fig. 1). However, the two experiments probe different physical states of the material. The highly emissive pristine powder corresponds to a well-ordered crystalline phase whose molecular conformation has been determined by single-crystal X-ray diffraction and is supported by computational studies as the lowest-energy conformational minimum. In contrast, rapid aggregation during the THF/water co-solvent experiments is expected to produce poorly ordered or amorphous aggregates rather than the thermodynamically favoured crystalline phase. Computational studies indicate the existence of multiple energetically accessible conformations for compound 2, suggesting that aggregation may kinetically trap an alternative conformational and packing state with substantially weaker emission. This interpretation is consistent with the mechanochromic studies, where disruption of the crystalline phase likewise produces a weakly emissive state that does not readily recover following solvent fuming. Therefore, the reduced fluorescence observed during the co-solvent experiments most likely reflects emission from kinetically trapped amorphous aggregates rather than the highly emissive crystalline phase.

Overall, these results demonstrate that constitutional isomerism not only governs the electronic structure of the fused TPE–carbazole framework but also controls the accessible conformational landscape and resulting solid-state organisation. These factors collectively determine whether aggregation suppresses or enhances non-radiative decay pathways, thereby dictating both the AIE response and the subsequent mechanochromic behaviour of the materials.

To investigate the variation in absorption wavelength, solvatochromic experiments were carried out compounds 1 and 2 by running the UV/Vis absorption in a wide range of solvents with varying polarity (Fig. S14). The solvatochromic response of both materials did not correlate with bulk solvent dielectric properties or macroscopic polarity scales, but instead aligned closely with specific microscopic solute–solvent interactions. The UV-vis absorption *λ*_max_ of both compounds exhibited a significant bathochromic shift across the evaluated solvent series, progressing from a minimum wavelength in *N*,*N*-dimethylformamide (DMF) to a maximum wavelength in chloroform. 11 nm and 17 nm for compound 1 and 2 respectively. The systematic red-shift observed in dichloromethane (DCM) and chloroform directly tracks the hydrogen-bond donating acidity of these chlorinated solvents. Because the chromophore features a conjugated carbonyl moiety, this behaviour indicates a specific hydrogen-bonding interaction at the carbonyl oxygen. The localized solvent-to-solute proton donation selectively stabilizes the highly polar π* excited state relative to the ground state, effectively shifting the absorption maximum to longer wavelengths. The larger solvatochromic shift observed for compound 2 indicates a more polar excited state that interacts more strongly with its local environment leading to greater variation in UV/Vis absorption compared with compound 1.

### 2.4. Mechano-responsive properties

The solid-state mechanoresponsive properties of all compounds were examined. Compounds 1 and 2 both exhibited significant changes in colour and fluorescence intensity upon the application of mechanical force, whereas compounds 3 and 4 did not. Compounds 1 and 2 were investigated in more detail. Pristine powder samples were placed between two glass slides, and their fluorescence spectra were recorded. The samples were then mechanically ground, followed by subsequent fluorescence measurements (Fig. 5).

**Fig. 5:**
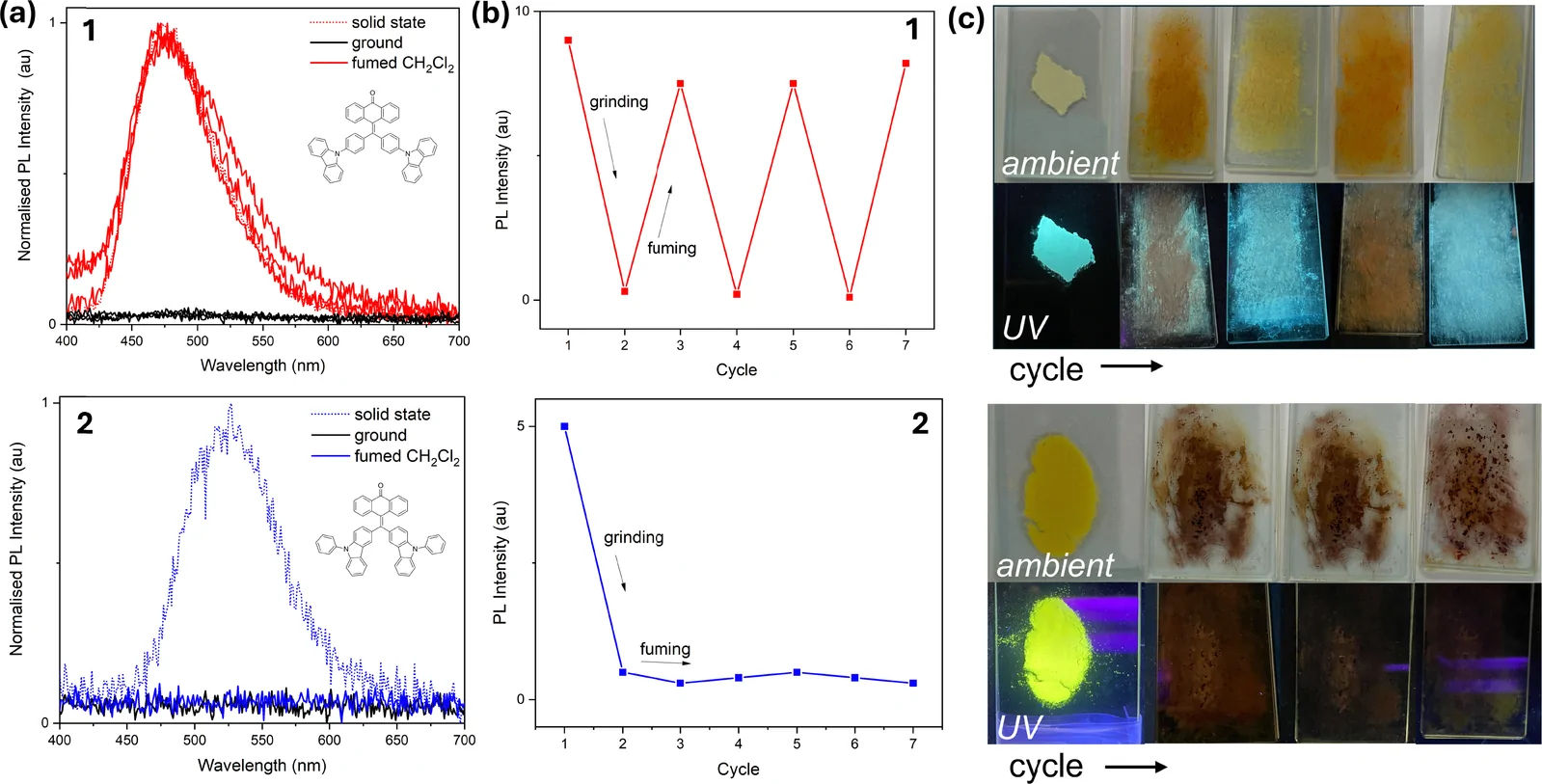
Mechanoresponsive fluorescent response of 1 and 2: (a) normalised PL spectra upon grinding and fuming, (b) PL intensity over 7 grinding and fuming cycles, (c) images taken under ambient light and 365 nm UV lamp illumination. Solid samples were placed between two glass slides and fluorescence intensity was measured before and after grinding mechanically or fuming with dichloromethane.

In its pristine state, compound 1 appears off-white and displays strong blue fluorescence with an emission maximum at 470 nm. Upon mechanical grinding, the fluorescence is significantly quenched, and the powder undergoes a visible colour change to rust orange. Exposure of the ground sample to dichloromethane vapour for 30 s restores both the original appearance and fluorescence intensity at 470 nm. This process was repeated over seven cycles, demonstrating the reversibility of the mechanochromic behaviour of 1.

In contrast, the mechanochromic response of compound 2 was found to be irreversible. The pristine solid exhibits a characteristic yellow colour and strong fluorescence with an emission maximum at 525 nm. Mechanical grinding results in fluorescence quenching accompanied by a colour change from yellow to red. Subsequent exposure to vapours of various solvents, including dichloromethane, methanol, toluene, and ethyl acetate, for extended periods of up to 1 hour did not induce recovery to the original emissive state. The yellow solid was only regenerated through fully redissolving in dichloromethane and precipitating into a counter solvent such as hexane.

In both cases, the observed mechanoresponsive behaviour is attributed to disruption of molecular packing and changes in intermolecular and intramolecular interactions. For 1 and 2, mechanical stimulation induces a red-shift, which is likely associated with enhanced π–π interactions arising from pressure-induced molecular planarisation. The notable difference in reversibility is attributed to a lower energetic barrier for structural reorganisation in 1 compared to 2. For compound 1, small amounts of solvent vapour diffusing into the material is sufficient for the reorganisation at ambient temperature, whereas 2 forms a more stable and resistant state.

FT-IR and PXRD studies were carried out to compare and characterise the difference between pristine and ground samples of 1 and 2 (Fig. 6). For compound 1, in the pristine state two weak peaks are observed between 1815 and 1690 cm^−1^ which do not appear in the ground sample spectra. A shift in relative intensities of the peaks at 1660 and 1595 cm^−1^ which correspond to the aromatic vibrations is also observed. Additional peaks are observed for compound 2 in the ground sample spectrum, notably at 1733, 2847 and 2819 cm^−1^. A shift in relative intensities of the peaks at 1664 and 1595 cm^−1^ and a general broadening is also observed. Powder X-ray diffraction (PXRD) analysis reveals that the pristine samples of compounds 1 and 2 exhibit well-defined, sharp diffraction peaks characteristic of a crystalline phase. Upon mechanical grinding of compound 1, the diffractogram shows a reduction in the relative intensities of peaks, namely at 6.5° and 11.5°, as well as subtle changes in minor reflections. This is consistent with a decrease in crystallite size and a loss of the preferred orientation. In addition to the reduction in peak intensities and the changes in reflection pattern of compound 2 upon grinding, there is notably a broad diffraction feature underlying the pattern centred around 18°. These changes are consistent not only with smaller crystallite size, but also with a reduction in long-range order in the material. Exposure of the ground powder of compound 1 to dichloromethane vapour suggests a restoration of this preferred orientation as evidenced by the reappearance of the sharper diffraction peaks and a pattern closer to that of the pristine material. In contrast, vapour fuming of compound 2 does not induce structural recovery. The difference in diffraction patterns between the pristine and ground samples is consistent with conformational change.

**Fig. 6:**
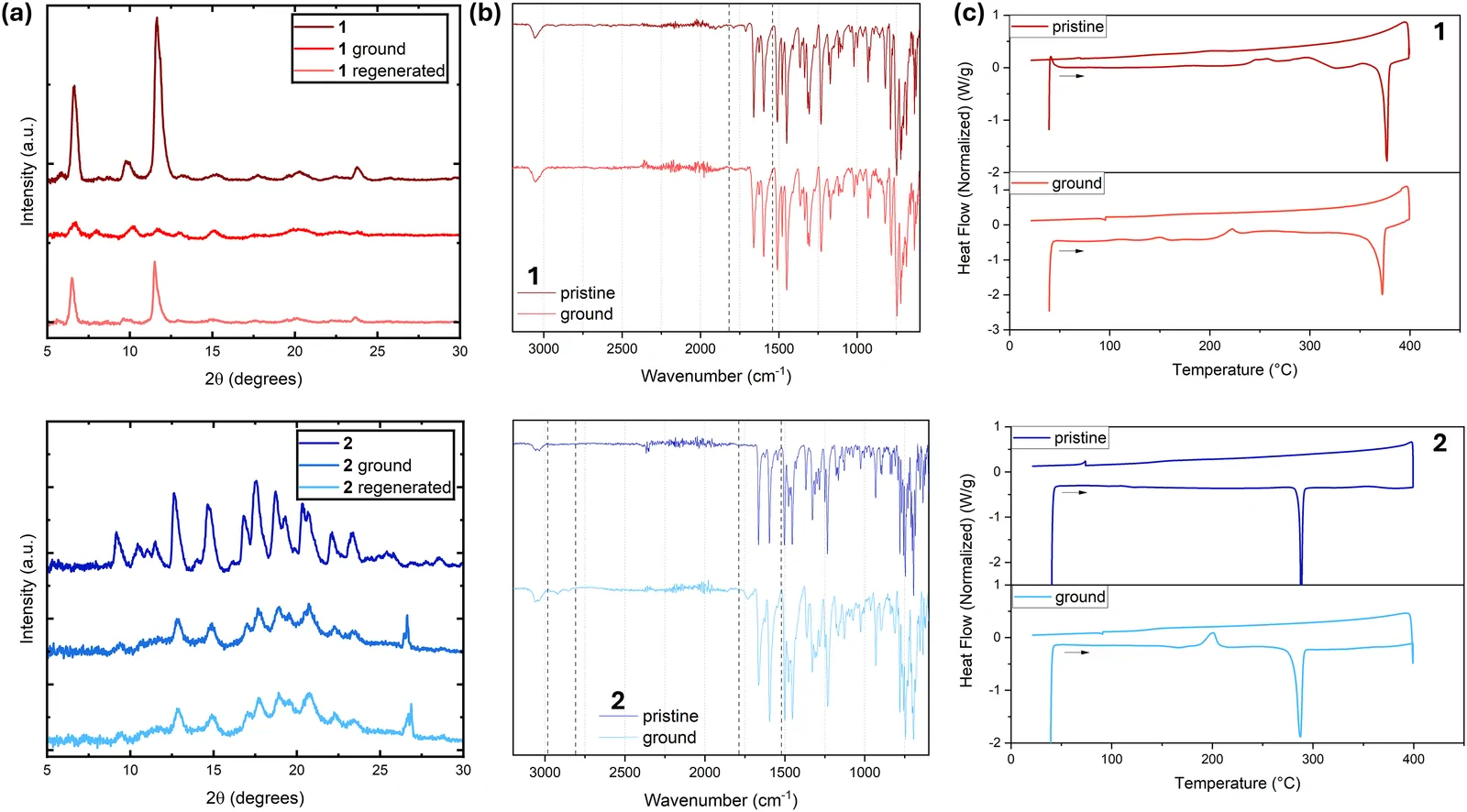
(a) PXRD spectra of 1 and 2 as pristine sample, after grinding and, after fuming with dichloromethane (regenerated), (b) IR spectra of 1 and 2 before and after grinding, and (c) DSC spectra of compound 1 and 2, before and after grinding.

Thermal analysis was carried out on compound 1 and 2. Differential scanning calorimetry revealed variations in phase transitions between pristine and ground samples (Fig. 6). For compound 1 in pristine form, two small peaks at 245 °C and 300 °C are present. In the ground form, a stronger exothermic peak at 230 °C is present corresponding to reorganisation of molecular packing into more crystalline state. Both samples show clear and obvious melt at 375 °C. For compound 2, both pristine and ground forms have obvious sharp melt at 290 °C. The ground sample displays a large exothermic peak at 200 °C which is absent from the pristine sample. This peak is attributed to a phase transition from amorphous to more crystalline. Thermal gravitational analysis revealed excellent thermal stability of both materials up to at least 400 °C, desirable for implementing materials into real world applications (Fig. S13).

Collectively, the PXRD, IR, and DSC measurements indicate that mechanical stimulation disrupts the ordered crystalline packing of compounds 1 and 2, producing a more disordered solid-state phase that is accompanied by fluorescence quenching. For compound 1, recovery of the diffraction pattern following solvent fuming is consistent with restoration of both the crystalline phase and fluorescence, whereas the absence of comparable recovery for compound 2 reflects its irreversible structural transformation. When considered together with the pressure-dependent fluorescence microscopy measurements, these complementary results support the proposed relationship between structural reorganisation and the observed optical response. Although the present study does not include *in situ* pressure-dependent diffraction measurements capable of quantitatively monitoring the evolution of crystallinity with applied pressure, the combined *ex situ* structural characterisation and pressure-dependent fluorescence data provide consistent evidence that pressure-induced disruption of the crystalline packing underpins the mechanochromic behaviour of these materials.

To investigate the origin of the contrasting mechanochromic reversibility between compounds 1 and 2, computational studies were performed (Fig. 7). Two-dimensional torsional scans revealed distinct conformational energy landscapes for the two materials. Compound 1, which exhibited reversible colour recovery upon solvent fuming, possessed two comparably stable conformational minima separated by a relatively low energy barrier. In contrast, compound 2, which displayed an irreversible mechanochromic response, exhibited two corresponding minima separated by a substantially higher energy barrier. Time-dependent DFT (TD-DFT) calculations performed for each minimum reproduced the experimentally observed spectral behaviour, with the alternative conformer displaying a red-shifted absorption consistent with the colour change observed after mechanical grinding. These results suggest that the as-prepared crystalline material preferentially adopts the conformation favoured by the original crystal packing, whereas mechanical grinding disrupts this packing and enables relaxation into an alternative conformational minimum associated with the red-shifted optical response. For compound 1, the relatively low reverse barrier facilitates recovery of the initial conformation during solvent-vapour fuming, while the larger barrier calculated for compound 2 promotes kinetic trapping of the mechanically generated state. This is highlighted in the 2D conformational map, where a continuous dark blue area with energy barrier no more than 1 kcal mol^−1^ connects both conformations of compound 1. The energy minima areas of compound 2 are separated by larger barrier of approximately 10 kcal mol^−1^ depicted by cyan area on 2D conformational map. Together, these findings support a mechanism in which mechanical stimulation induces conformational reorganisation, with the contrasting reversibility arising from differences in the kinetic accessibility of the reverse conformational pathway.

**Fig. 7:**
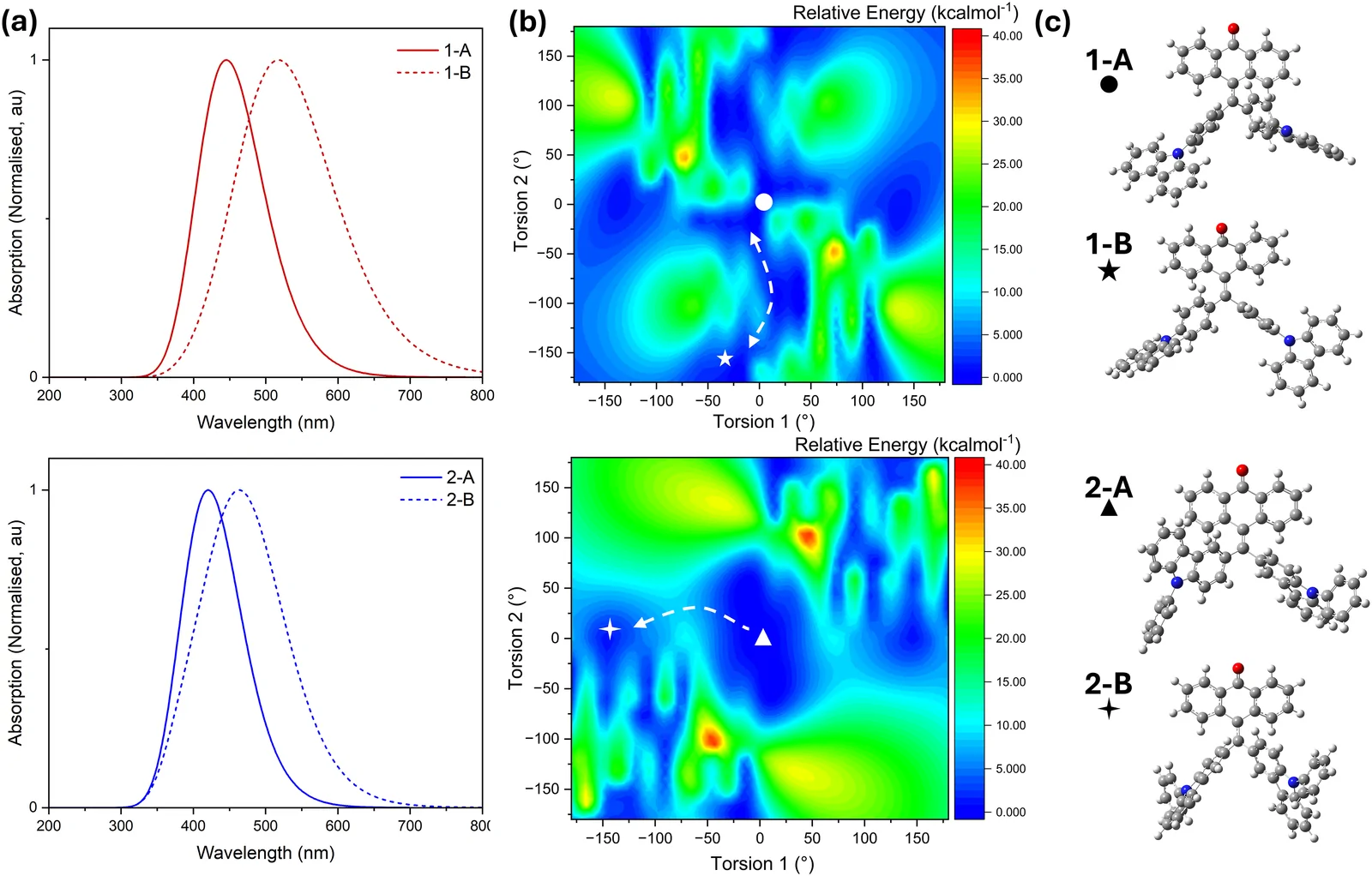
(a) TD-DFT predicted UV/Vis absorption spectra for conformers 1-A, 1-B, 2-A, 2-B, and (b) 2D torsional scan of 1 and 2. The torsions measured are between central alkene and phenyl rings of *N*-phenylcarbazole units. Lowest energy conformation 1-A and 2-A are set to 0°,0° and rotation away from this point was recorded around 180° in both directions in 15° increments. The geometry was optimised at each step and energy calculated. Rotational pathways between low energy conformations are highlighted by arrows. (c) Optimised geometry of lowest energy conformers of 1 and 2 (1-A, 1-B, 2-A, 2-B) corresponding to local minima on the 2-dimensional potential energy scan. All calculations carried out using Gaussian 16 using DFT B3LYP functional with the 6-311G(d,p) basis set.

To quantify the mechanochromic response to pressure of compound 1, paper films were dip-coated in dichloromethane solution of compound 1 and exposed to incrementally increasing pressure applied using a torque wrench press. A 2 mm^2^ cross-section of the films were imaged using a fluorescent microscope exciting the film at 410 nm, then measuring fluorescence intensity between 415–530 nm (Fig. 8). It was found that the fluorescence intensity dropped off sharply at around 0.2 MPa of pressure and was completely quenched at pressure of 0.9 MPa. The fluorescence quenching is consistent with pressure-induced disruption of the crystalline packing, as supported by the *ex situ* PXRD, IR, and DSC analyses. A pressure of 0.2 MPa corresponds to low-level mechanical stimuli readily generated by manual handling or gentle impact events, highlighting the potential of this material for low-force impact or touch sensing applications.

**Fig. 8:**
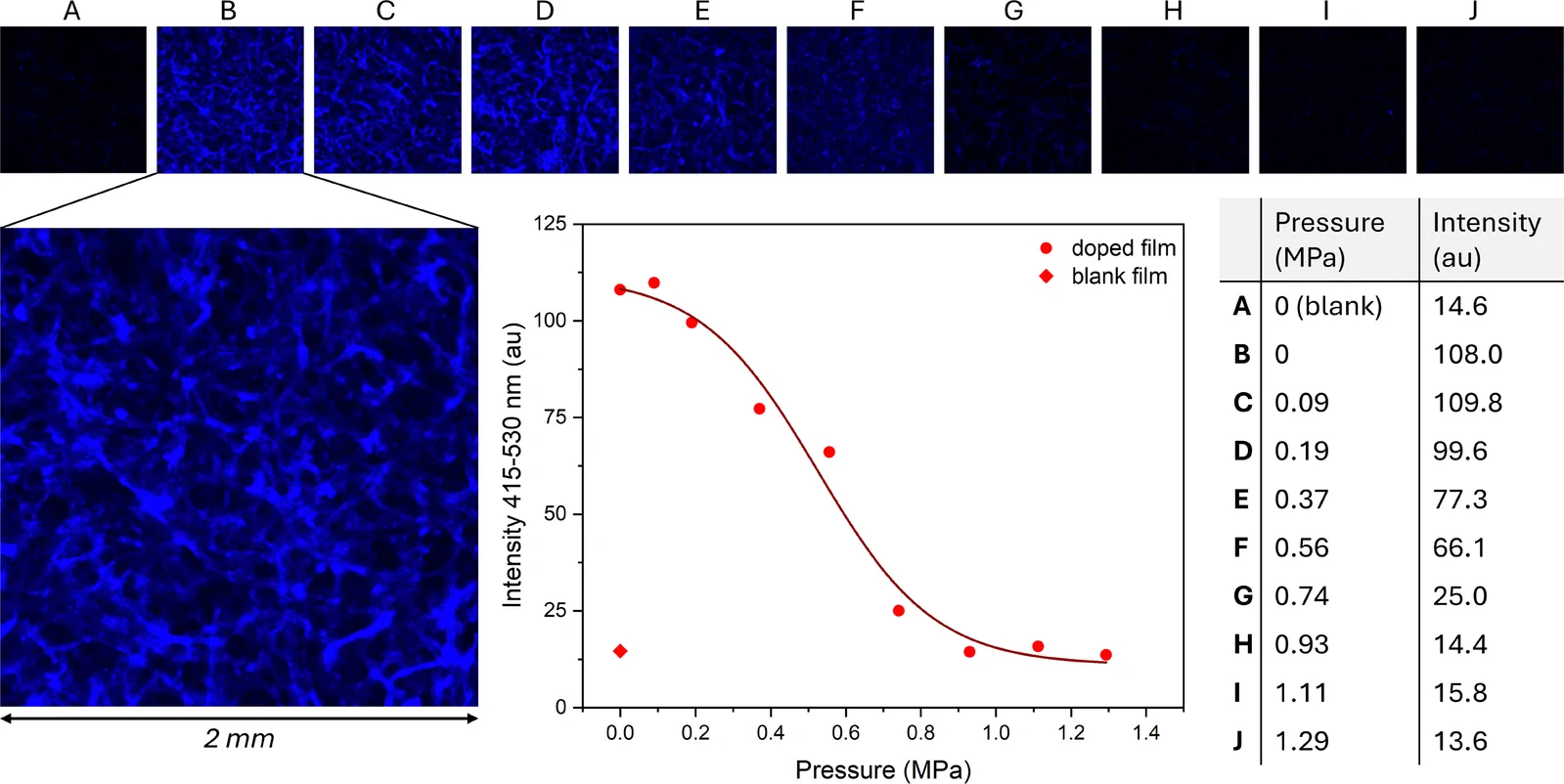
Quantifying the mechanofluorescence in relation to pressure for compound 1 using fluorescence microscopy to image paper substrates under incremental pressure increase. Excitation at 410 nm and fluorescence detected between 415–530 nm. Images A–J correspond to pressures of 0–1.29 MPa pressure. Intensity of pixels averaged over 2 mm^2^ cross section. Images are pseudocoloured blue with brightness representing light intensity detected between 415–530 nm.

### 2.5. Encrypted information transfer

To demonstrate the highly reversible and sensitive mechanochromic behaviour of 1, experiments were carried out to apply the compound as an invisible ink. Paper films were coated with compound 1*via* dip coating in dichloromethane solutions, and then dried. A shape was then imprinted into the film using a stamp or drawn onto the film using an inkless ballpoint pen to leave an imprint only visible under UV illumination (Fig. 9). The invisible ink could be erased by briefly exposing the paper film to dichloromethane vapour for 10 s, regenerating the pristine emissive state and enabling repeated reuse. The imprinting process was sufficiently gentle that the written features remained invisible under ambient light, while becoming clearly detectable only upon UV excitation. This approach enables the storage of information on low-cost, flexible substrates with a high degree of concealment, offering potential applications in anti-counterfeiting technologies, secure information encoding, temporary data storage, and tamper-evident labelling. Moreover, the use of solution-processable materials and simple vapour-treatment protocols suggests potential scalability, while future efforts may focus on replacing volatile organic solvents with more environmentally benign alternatives.

**Fig. 9:**
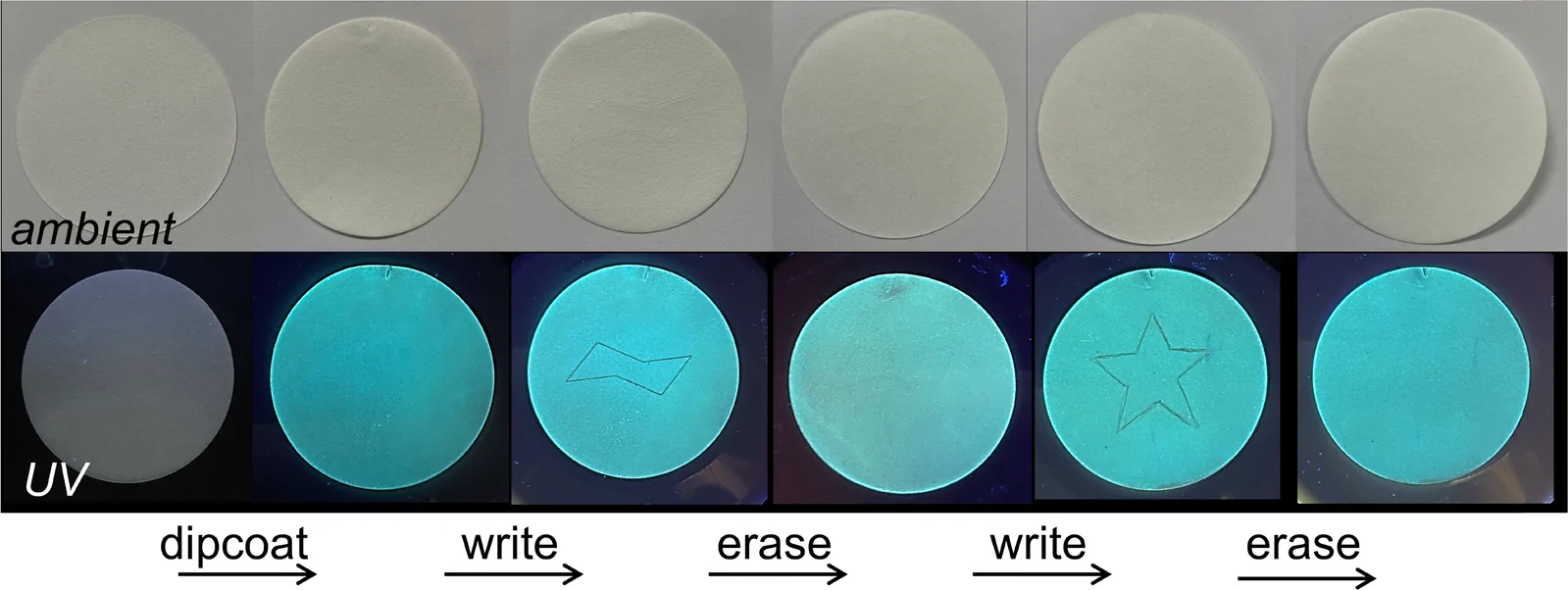
Demonstration of compound 1 as invisible ink. Paper substrates were dip coated in 1 × 10^−3^ mol L^−1^ dichloromethane solution, and then dried under ambient conditions for 0.5 h. Writing performed *via* stamping with 3D printed stamp (chair conformation) or writing with inkless ballpoint pen (star). Erasing performed by suspending film above a beaker of dichloromethane for 10 seconds. Films photographed under ambient light and under 365 nm UV lamp.

## 3. Conclusion

Four constitutional isomeric fused TPE–carbazole compounds (1–4) were successfully synthesised, providing a homologous molecular platform for systematically investigating the influence of isomeric connectivity on optoelectronic, aggregation-induced emission (AIE), and mechanoresponsive properties. While compounds 1, 3, and 4 exhibited AIE behaviour, only compounds 1 and 2 displayed solid-state mechanochromism, demonstrating that subtle variations in molecular connectivity can profoundly influence pressure-responsive behaviour.

Detailed XRD, IR, thermal and computational analyses revealed that the mechanochromic responses of compounds 1 and 2 originate from pressure-induced disruption of their crystalline packing, leading to a transition from one solid state to another accompanied by fluorescence quenching. Notably, compound 1 exhibited highly reversible mechanochromism over multiple grinding–fuming cycles, whereas compound 2 underwent an irreversible transition unless recrystallised, due to difference in reorganisational energy barriers, highlighting the critical role of molecular connectivity in governing reversibility.

Fluorescence imaging further demonstrated that compound 1 possesses an exceptionally low pressure-sensing threshold of 0.2 MPa, enabling optical detection of gentle mechanical stimuli generated by routine handling or light contact. This ultra-low activation pressure, together with its excellent reversibility, makes compound 1 particularly attractive for low-force pressure sensing, touch-responsive devices, and reusable optical information encryption applications.

Overall, this work establishes clear structure–property relationships linking constitutional isomeric connectivity to electronic structure, solid-state packing, aggregation behaviour, pressure sensitivity, and mechanochromic reversibility in fused TPE–carbazole systems. These findings not only provide molecular design principles for developing highly sensitive and reversible organic piezochromic materials but also demonstrate the value of systematic isomeric studies for advancing next-generation stimulus-responsive functional materials.

## Author contributions

DW wrote the manuscript, designed and synthesised the molecules, and carried out optoelectronic, electrochemical and computational characterisation. PXRD was carried out and analysed by MM and PD. AWS carried out additional characterisation experiments. SCXRD was carried out by SKD and CW. DW and GC conceived the experiments and project idea. GC, PD and MK supervised the project and provided guidance. All authors participated in discussing the results, manuscript writing and approved the final submission.

## Conflicts of interest

There are no conflicts to declare.

## Supplementary Material

TC-014-D6TC01521B-s001

TC-014-D6TC01521B-s002

## Data Availability

All data supporting the findings of this study are available within the article and supplementary information (SI). Supplementary information includes molecular characterisation data (NMR, mass spec), additional thermal, optical and computational data, and X-ray diffraction data. See DOI: https://doi.org/10.1039/d6tc01521b. Data will be available upon request from the authors.
