# Supplementary material for: Molecular connectivity–driven tuning of aggregation-induced emission and mechanoresponsive properties in fused TPE–carbazole materials
Source: J Mater Chem C Mater. 2026 Aug 21;14(38):18154–64. doi: 10.1039/d6tc01521b (PMC13648121; doi:10.1039/d6tc01521b)
Supplement: Supplementary file 1 [file TC-014-D6TC01521B-s001.pdf]

Supplementary Information (SI) for Journal of Materials Chemistry C.
This journal is © The Royal Society of Chemistry 2026

Supporting Information

Molecular connectivity–driven tuning of aggregation-induced emission and mechanoresponsive properties in fused TPE–carbazole materials

Dylan Wilkinson [a], Madeleine McRoberts [a], Abdul-Wasir Shaka [a], Sarah K. Dugmore [a], Claire Wilson [a], Pablo Docampo [b], Malcolm Kadodwala [a], Graeme Cooke [a]**

a. *School of Chemistry, University of Glasgow, University Place, Glasgow, G12 8QQ, UK.*

b. *Basque Center for Materials, Applications and Nanostructures (BCMaterials), 48940 Leioa, Spain.*

** Corresponding authors*

Contents

Supporting Information 1
1. General Methods 3
2. Synthesis 4
3. NMR Spectra 7
4. Mass Spectra 11
5. Thermal Analysis 13
6. Solvatochroism 14
7. Computational 15
8. X-ray Crystal Data 16
9. Reference 23

1. General Methods

Reagents and solvents were purchased from either Merck® or Fluorochem®. TLCs were performed using Merck silica gel 60 covered aluminium plates F254. Reaction temperatures: room temperature refers to 20–25 °C.

Mass spectrometry and elemental analysis were obtained from the mass spectrometry service at the University of Glasgow. NMRs were recorded on either Bruker Avance III 400. The ^{1}H and ^{13}C spectra were recorded with TMS as internal standard. NMR spectra were analysed using MNova®. Signals in ^{1}H NMR spectra are described as singlet (s), doublet (d), triplet (t), quartet (q), quintet (quint), multiplet (m), broad (b) or a combination of these, which refers to the spin-spin coupling pattern observed.

Absorbance spectrometry was performed using a Shimadzu UV-3600 UV-Vis-NIR spectrophotometer. Qualitative fluorescence spectroscopy was performed using a Shimadzu RF-5301PC spectrofluorimeter. All the spectroscopic data was processed using Origin Pro 8.5 software suite.

Electrochemical measurements were conducted in dichloromethane solutions (5×10^{-4} mol L^{-1}) containing tetrabutylammonium tetrafluoroborate (TBAB, 0.1 mol L^{-1}) as the supporting electrolyte. All experiments were performed at room temperature under thoroughly degassed conditions.

Fluorescence imaging was carried out on a Zeiss LSM 710 confocal fluorescence microscope system using an upright Axio Imager 2 microscope with 405 nm diode laser with CW excitation. For mechanochromic sensing experiments, pressure was applied to films using a torque wrench mechanical press.

Thermogravimetric analysis was carried out on a Netzsch TG 209 F3 Tarsus Thermogravimetric Analyser. The experiments were run under a flow of nitrogen with a heating rate of 10 K min^{-1} between 25 °C – 550 °C. Differential scanning calorimetry was carried out on a Netzsch DSC 214 Polyma instrument under nitrogen. The scans were carried out at a heating/cooling rate of 10 K min^{-1} with the maximum temperature 400°C.

PXRD analysis was carried out on a Rigaku MiniFlex benchtop diffractometer equipped with Cu sealed tube X-ray source.

Computational calculations were performed using Gaussian '16 software suite.[1] Molecular geometries were initially optimized semi-empirically (PM6) and then reoptimized by DFT (B3LYP/6- 311G dp). Energy minima were confirmed by the absence of imaginary frequencies from vibrational frequency calculations.

2. Synthesis

10-Dibromomethylene-9-anthrone (**DMBA**) [2]

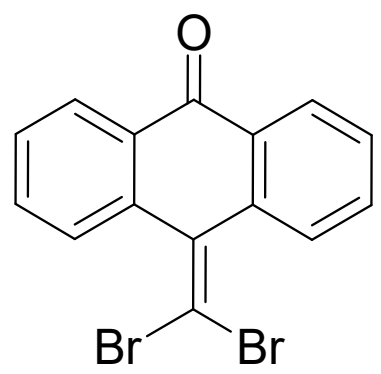

Anthraquinone (0.52 g, 2.50 mmol) and carbon tetrabromide (0.93 g, 2.8 mmol) were added to dry dichloromethane (100 mL) and the solution was cooled to 0 °C. Triethylphosphite (1.03 mL, 6.0 mmol) was added dropwise and the reaction stirred at room temperature for 12 h. The reaction mixture was washed with water (2 × 30 mL) and saturated ammonium chloride solution (2 × 30 mL). The combined aqueous phase was washed with dichloromethane (3 × 30 mL), dried over $MgSO_4$ then filtered. The solvent was removed in vacuo then the crude material was purified via flash column chromatography (SiO_2, dichloromethane/petroleum ether 3:2) to yield a white solid (0.25 g, 35%). ^{1}H NMR (400 MHz, $CDCl_3$): δ = 8.09 (m, 4H, Ar*H*), 7.55 (td, *J* = 6.8, 3.4 Hz, 2H, Ar*H*), 7.49 (td, *J* = 6.8, 4.0 Hz, 2H, Ar*H*); ^{13}C NMR (101 MHz, $CDCl_3$): δ = 185.3, 138.3, 136.8, 132.6, 131.1, 128.9, 128.6, 126.8, 93.7; MS (ESI+) m/z = 386.88 $[m+Na]^+$.

10-(Bis[4-(9H-carbazol-9-yl)phenyl]methylidene)-9,10-dihydroanthracen-9-one (**1**)

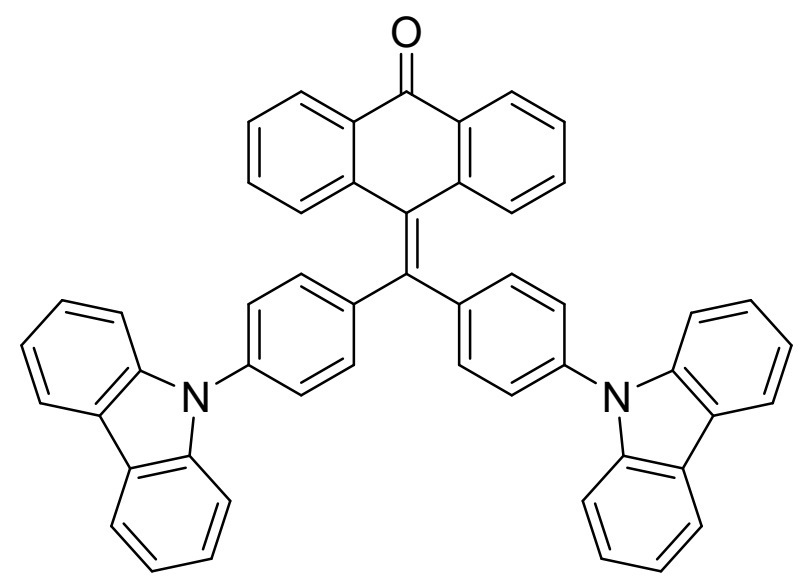

DMBA (0.10 g, 0.27 mmol), 4-(9H-carbozol-9-yl)phenylboronic acid (0.32 g, 1.1 mmol), potassium carbonate (0.37 g, 2.7 mmol) and tetrakis(triphenylphosphine)palladium (0.05 g, 0.05 mmol) were added to degassed tetrahydrofuran/water mixture (50 mL, 4:1). The reaction was heated to 75 °C for 24 h in a sealed tube. After cooling to room temperature, the reaction mixture was added to water (30 mL) and extracted with dichloromethane (3 × 30 mL), dried over $MgSO_4$ then filtered. The solvent was removed in vacuo then the crude material was purified via flash column chromatography (SiO_2, dichloromethane/petroleum ether 1:1) to yield a white solid (0.12 g, 67%) . ^{1}H NMR (400 MHz, $CDCl_3$): δ = 8.23 (dd, *J* = 7.8, 1.0 Hz, 2H, Ar*H*), 8.16 (d, *J* = 7.8 Hz, 4H, Ar*H*), 7.56 (s, 8H, Ar*H*), 7.47 – 7.40 (m, 10H, Ar*H*), 7.34 – 7.27 (m, 8H, Ar*H*); ^{13}C NMR (101 MHz, $CDCl_3$): δ = 185.9, 144.2, 141.7, 140.8, 139.3, 137.1, 133.4, 132.6, 131.0, 130.7, 129.4, 128.0, 127.5, 127.0, 126.2, 123.7, 120.6, 120.3, 109.8; MS (ESI+) m/z = 711.2373 $[m+Na]^+$ (calculated for $C_{51}H_{32}N_2NaO_1^+$: 711.2407).

10-[Bis(9-phenyl-9H-carbazol-2-yl)methylidene]-9,10-dihydroanthracen-9-one (**3**)

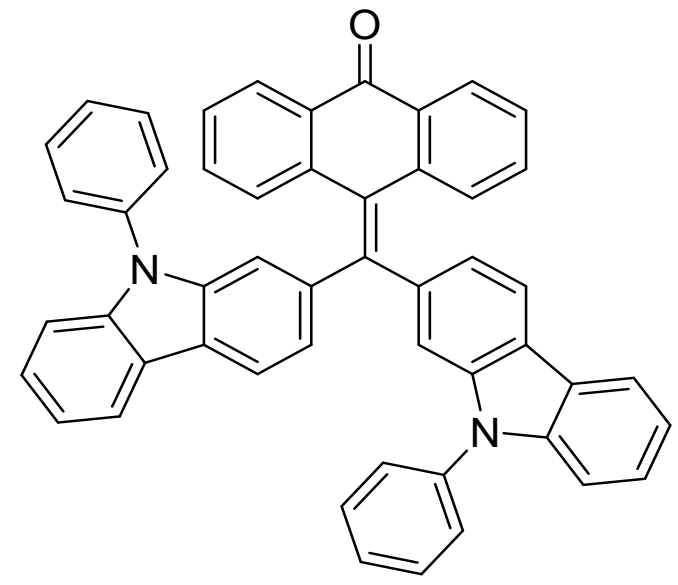

DMBA (0.06 g, 0.16 mmol), *N*-phenyl-9H-carbazol-2-boronic acid (0.14 g, 0.48 mmol), potassium carbonate (0.22 g, 1.6 mmol) and tetrakis(triphenylphosphine)palladium (0.04 g, 0.03 mmol) were added to degassed tetrahydrofuran/water mixture (20 mL, 4:1). The reaction was heated to 90 °C for 24 h in a sealed tube. After cooling to room temperature, the reaction mixture was added to water (15 mL) and extracted with dichloromethane (3 × 15 mL), dried over $MgSO_4$ then filtered. The solvent was removed in vacuo then the crude material was purified via flash column chromatography (SiO_2, toluene/petroleum ether 3:2) to yield a yellow solid (0.09 g, 87%). ^1H NMR (400 MHz, $CDCl_3$): δ = 8.21 (dd, *J* = 7.8, 1.2 Hz, 2H, Ar*H*), 8.08 (dd, *J* = 14.5, 7.9 Hz, 4H, Ar*H*), 7.54 – 7.45 (m, 4H, Ar*H*), 7.45 – 7.35 (m, 6H, Ar*H*), 7.33 – 7.26 (m, 6H, Ar*H*), 7.26 – 7.14 (m, 8H, Ar*H*), 7.06 – 6.97 (m, 2H, Ar*H*); ^{13}C NMR (101 MHz, $CDCl_3$) δ = 185.9, 148.3, 141.4, 141.0, 140.8, 140.2, 137.4, 133.1, 131.0, 130.8, 130.0, 129.5, 127.6, 127.1, 126.8, 126.5, 126.3, 123.2, 122.6, 121.8, 120.6, 120.5, 120.3, 111.5, 109.9; MS (ESI+) m/z = 711.2383 $[m+Na]^+$ (calculated for $C_{51}H_{32}NNaO^+$: 711.2407).

10-(Bis[3-(9H-carbazol-9-yl)phenyl]methylidene)-9,10-dihydroanthracen-9-one (**4**)

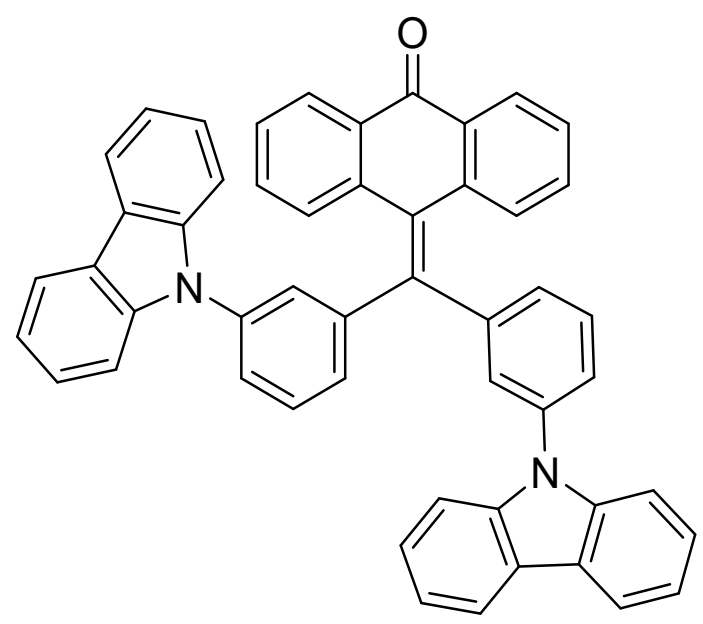

DMBA (0.06 g, 0.16 mmol), 3-(9H-carbazol-9-yl)phenylboronic acid (0.14 g, 0.48 mmol), potassium carbonate (0.22 g, 1.6 mmol) and tetrakis(triphenylphosphine)palladium (0.04 g, 0.03 mmol) were added to degassed tetrahydrofuran/water mixture (20 mL, 4:1). The reaction was heated to 90 °C for 24 h in a sealed tube. After cooling to room temperature, the reaction mixture was added to water (15 mL) and extracted with dichloromethane (3 × 15 mL), dried over $MgSO_4$ then filtered. The solvent was removed in vacuo then the crude material was purified via flash column chromatography (SiO_2, dichloromethane/petroleum ether 1:1) followed by recrystallisation in acetonitrile to yield a yellow solid (0.05 g, 46%). ^1H NMR (400 MHz, $CDCl_3$) δ = 8.27 (dd, *J* = 7.8, 1.0 Hz, 2H, Ar*H*), 8.11 – 8.04 (m, 4H, Ar*H*), 7.61 (t, *J* = 7.7 Hz, 2H, Ar*H*), 7.57 – 7.42 (m, 6H, Ar*H*), 7.37 (d, *J* = 7.3 Hz, 4H, Ar*H*), 7.34 – 7.29 (m, 2H,

Ar*H*), 7.29 – 7.18 (m, 8H, Ar*H*), 6.99 (m, 4H, Ar*H*); ^{13}C NMR (101 MHz, $CDCl_3$) δ = 185.5, 143, 143.8, 140.6, 139.1, 138.6, 133.5, 132.5, 131.2, 130.5, 129.3, 127.9, 127.8, 127.4, 127.2, 126.1, 125.8, 123.6, 120.5, 120.2, 109.6; MS (ESI+) m/z = 711.2407 $[m+Na]^+$ (calculated for $C_{51}H_{32}N_2NaO^+$: 711.2407).

10-[Bis(9-phenyl-9H-carbazol-3-yl)methylidene]-9,10-dihydroanthracen-9-one (**2**)

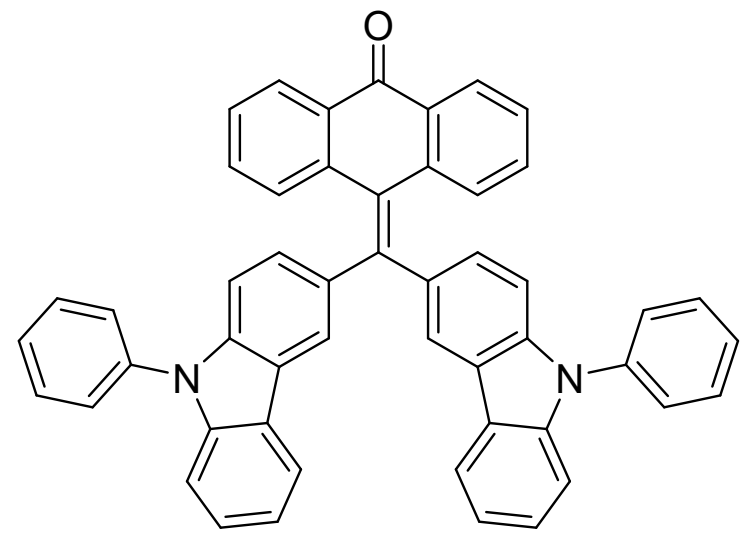

DMBA (0.08 g, 0.22 mmol), *N*-phenyl-9H-carbazol-3-boronic acid (0.19 g, 0.66 mmol), potassium carbonate (0.30 g, 2.2 mmol) and tetrakis(triphenylphosphine)palladium (0.05 g, 0.05 mmol) were added to degassed tetrahydrofuran/water mixture (5 mL, 4:1). The reaction was heated to 75 °C for 48 h. After cooling to room temperature, the reaction mixture was added to water (30 mL) and extracted with dichloromethane (3 × 30 mL), dried over $MgSO_4$ then filtered. The solvent was removed in vacuo then the crude material was purified via flash column chromatography (SiO_2, dichloromethane/petroleum ether 1:1) to yield a yellow solid (0.12 g, 80%). ^{1}H NMR (400 MHz, $CDCl_3$): δ = 8.22 (d, *J* = 7.6 Hz, 2H, Ar*H*), 8.05 (d, *J* = 7.7 Hz, 4H, Ar*H*), 7.57 (dt, *J* = 8.5, 7.5 Hz, 8H, Ar*H*), 7.49 – 7.43 (m, 2H, Ar*H*), 7.42 – 7.39 (m, 4H, Ar*H*), 7.38 – 7.34 (m, 2H, Ar*H*), 7.33 – 7.24 (m, 8H, Ar*H*), 7.02 (t, *J* = 7.3 Hz, 2H, Ar*H*); ^{13}C NMR (101 MHz, $CDCl_3$) δ = 186.3, 149.2, 141.4, 140.4, 140.2, 137.6, 136.2, 132.8, 130.9, 130.0, 129.5, 128.4, 127.7, 127.1, 127.0, 126.6, 126.3, 123.7, 123.4, 121.0, 120.7, 120.3, 110.1, 110.1; MS (ESI+) m/z = 689.2573 $[M]^+$ (calculated for $C_{51}H_{33}N_2O^+$: 689.2587).

3. NMR Spectra

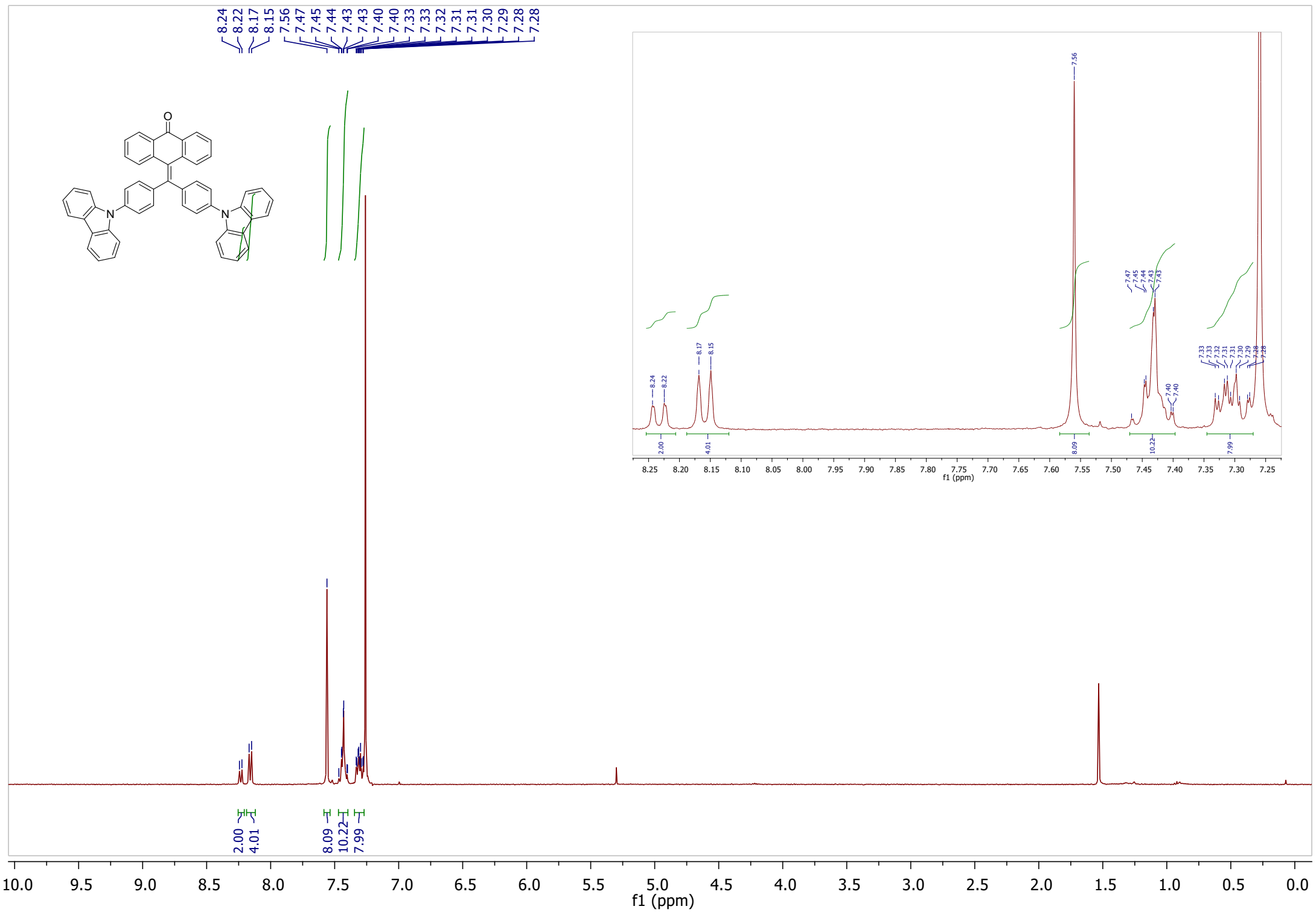

*Figure S1: Compound **1** ^{1}H NMR*

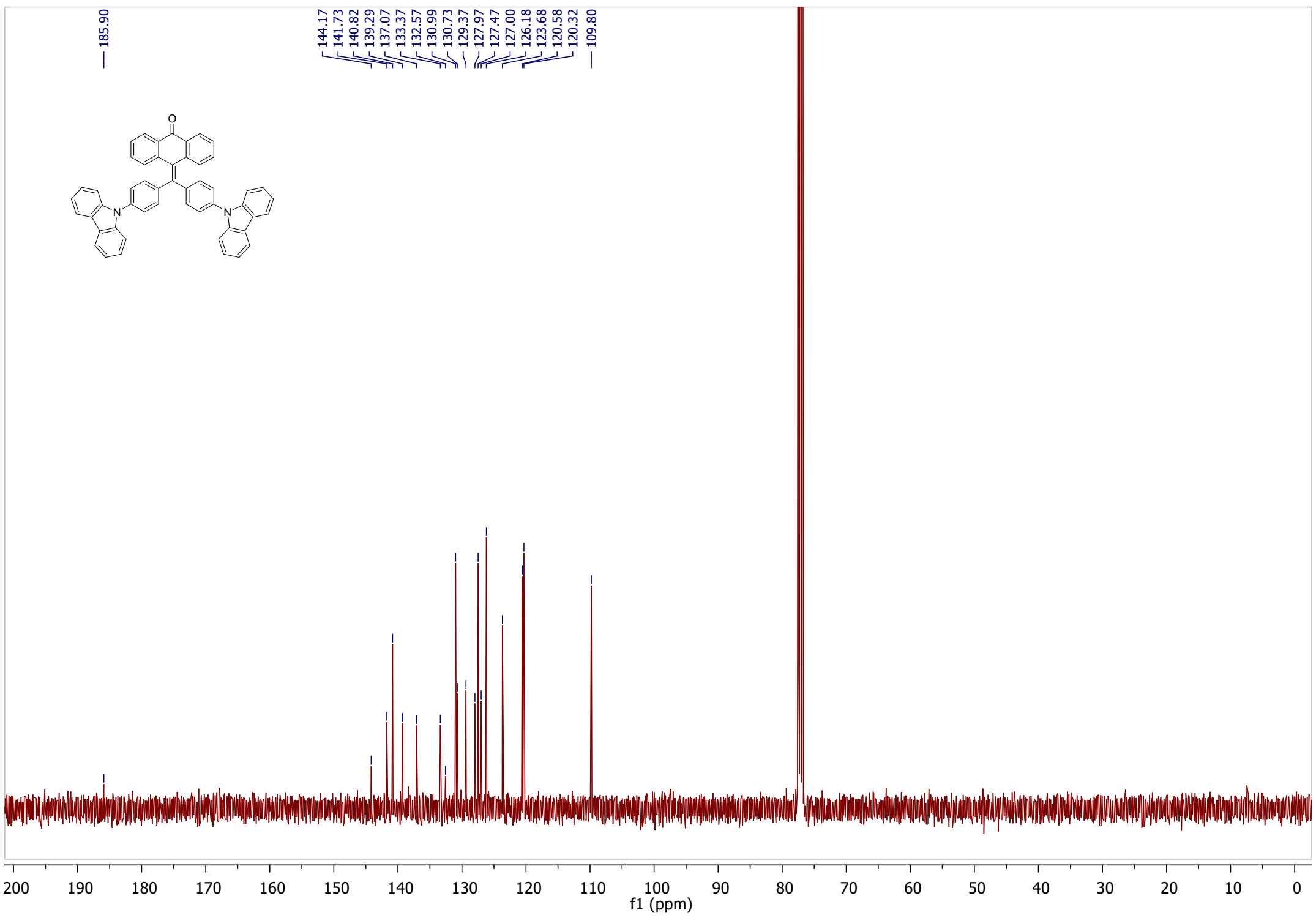

*Figure S2: Compound **1** ^{13}C NMR*

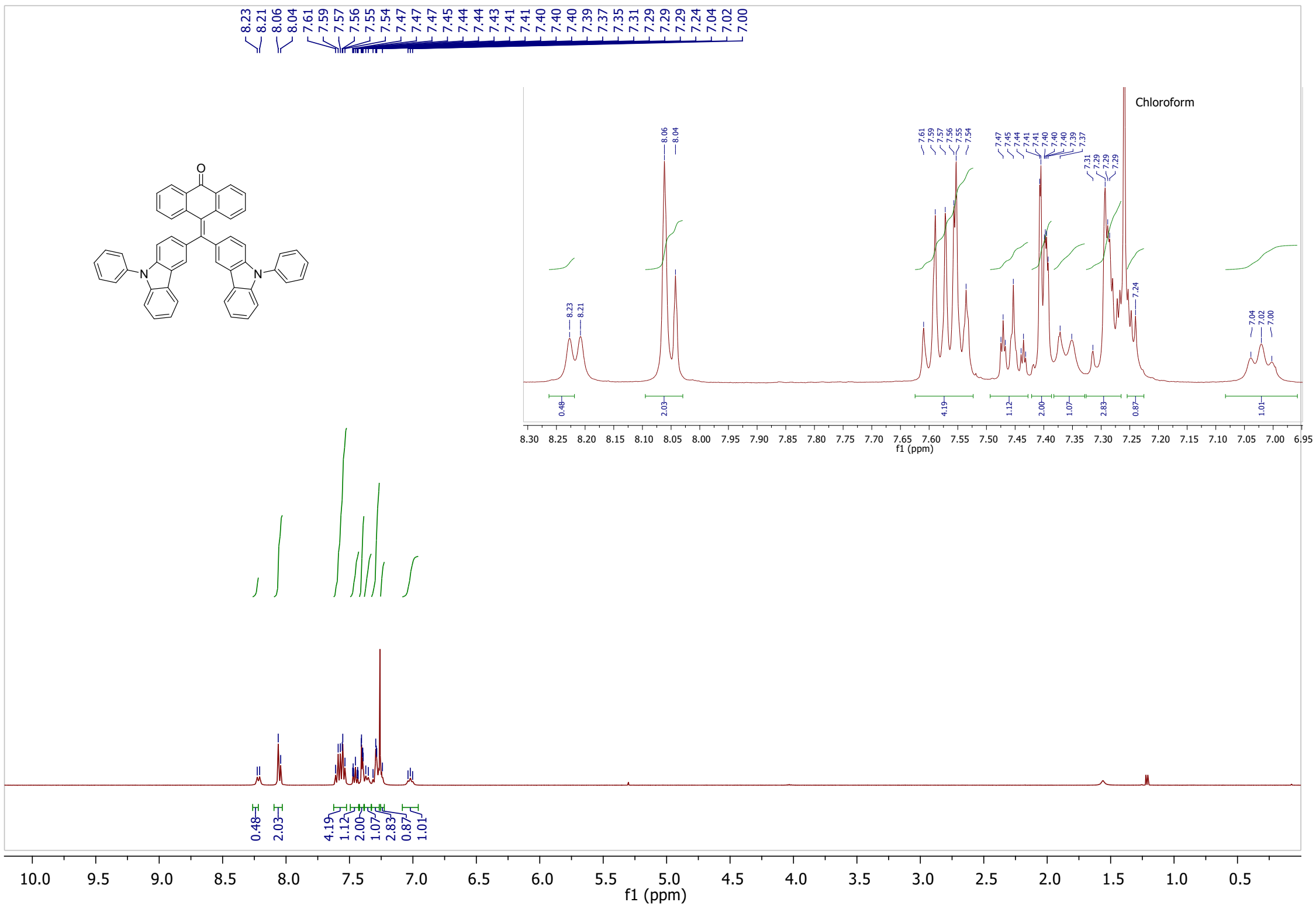

*Figure S3: Compound **2** ^{1}H NMR*

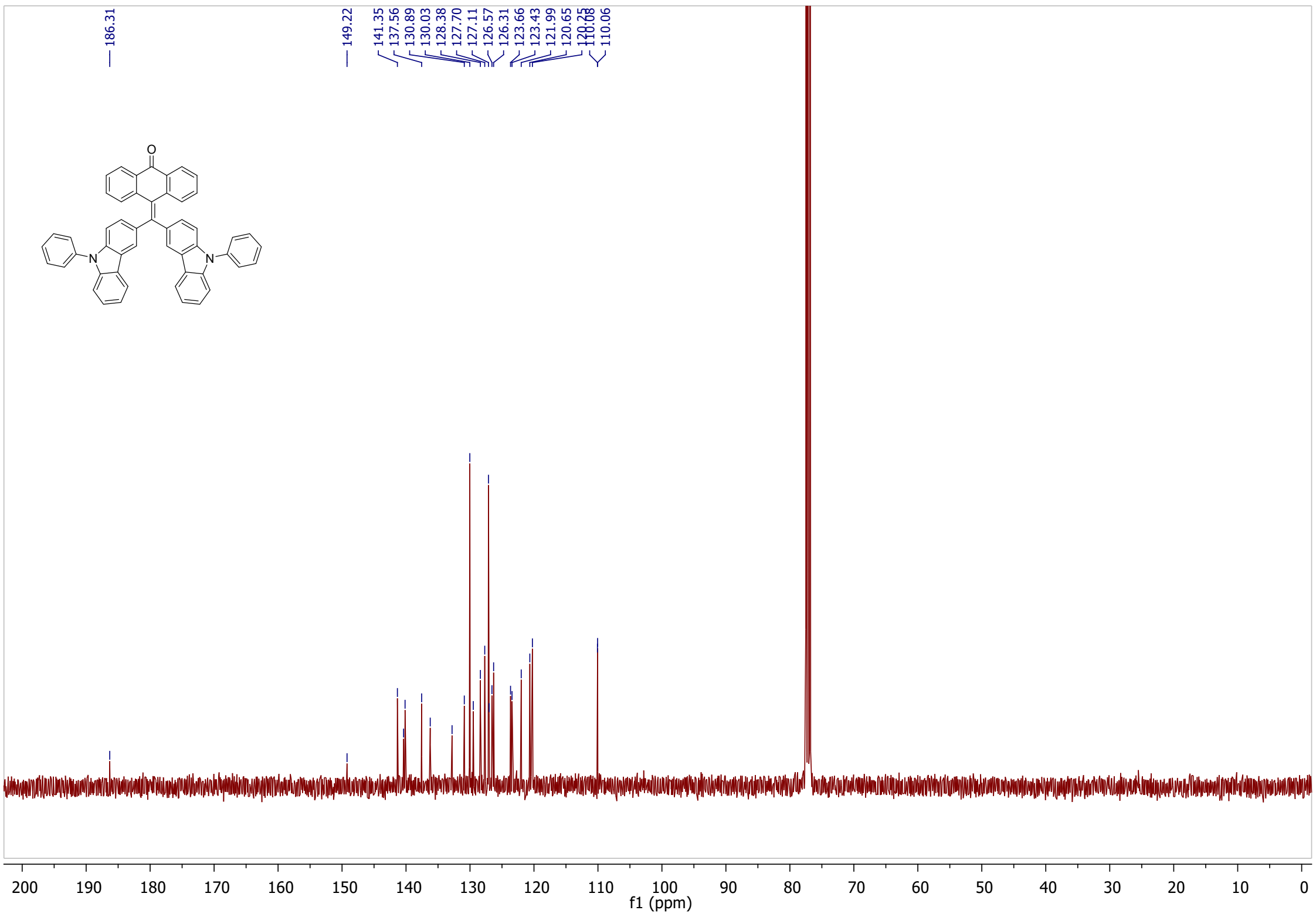

*Figure S4: Compound **2** ^{13}C NMR*

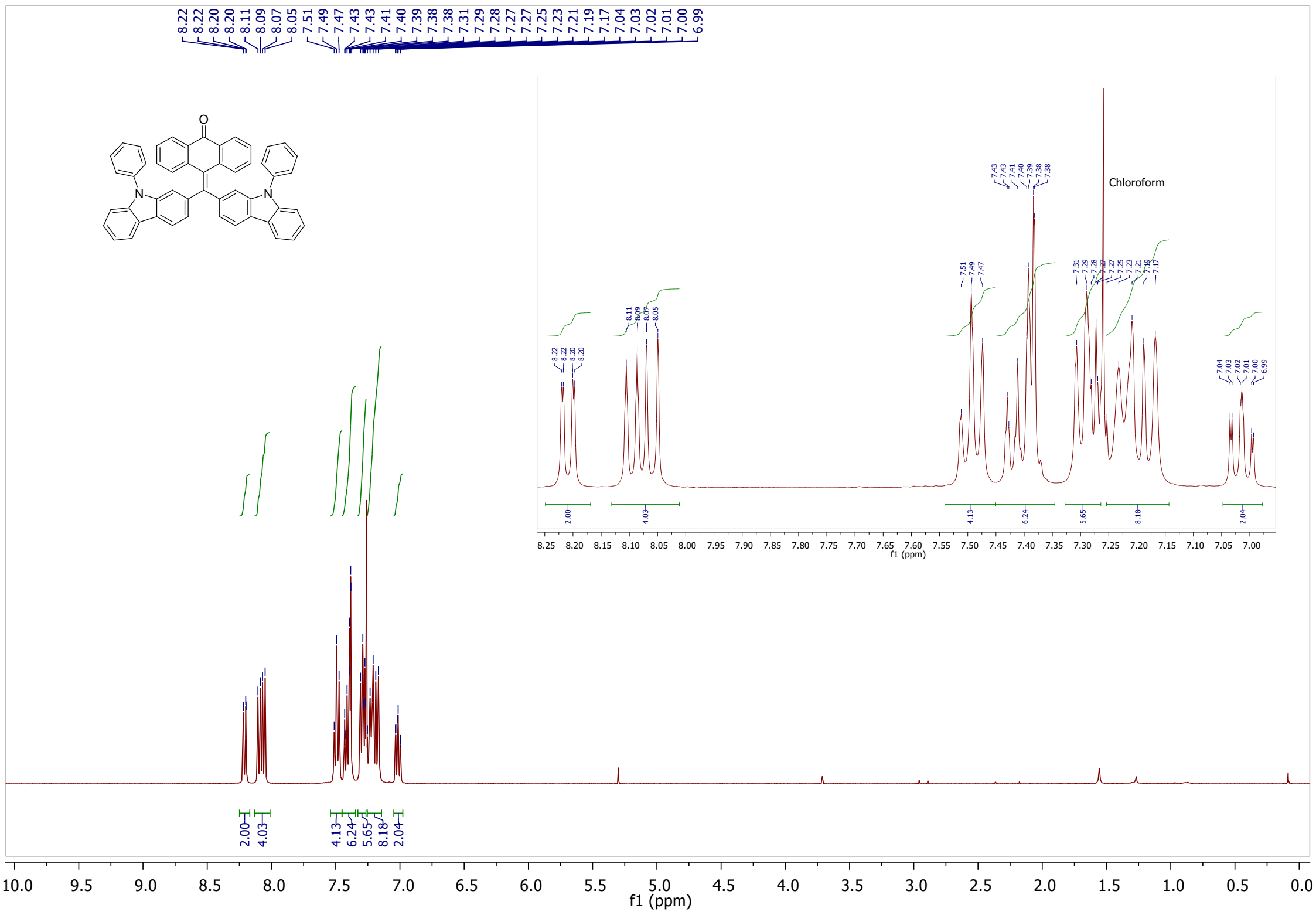

*Figure S5: Compound **3** ^{1}H NMR*

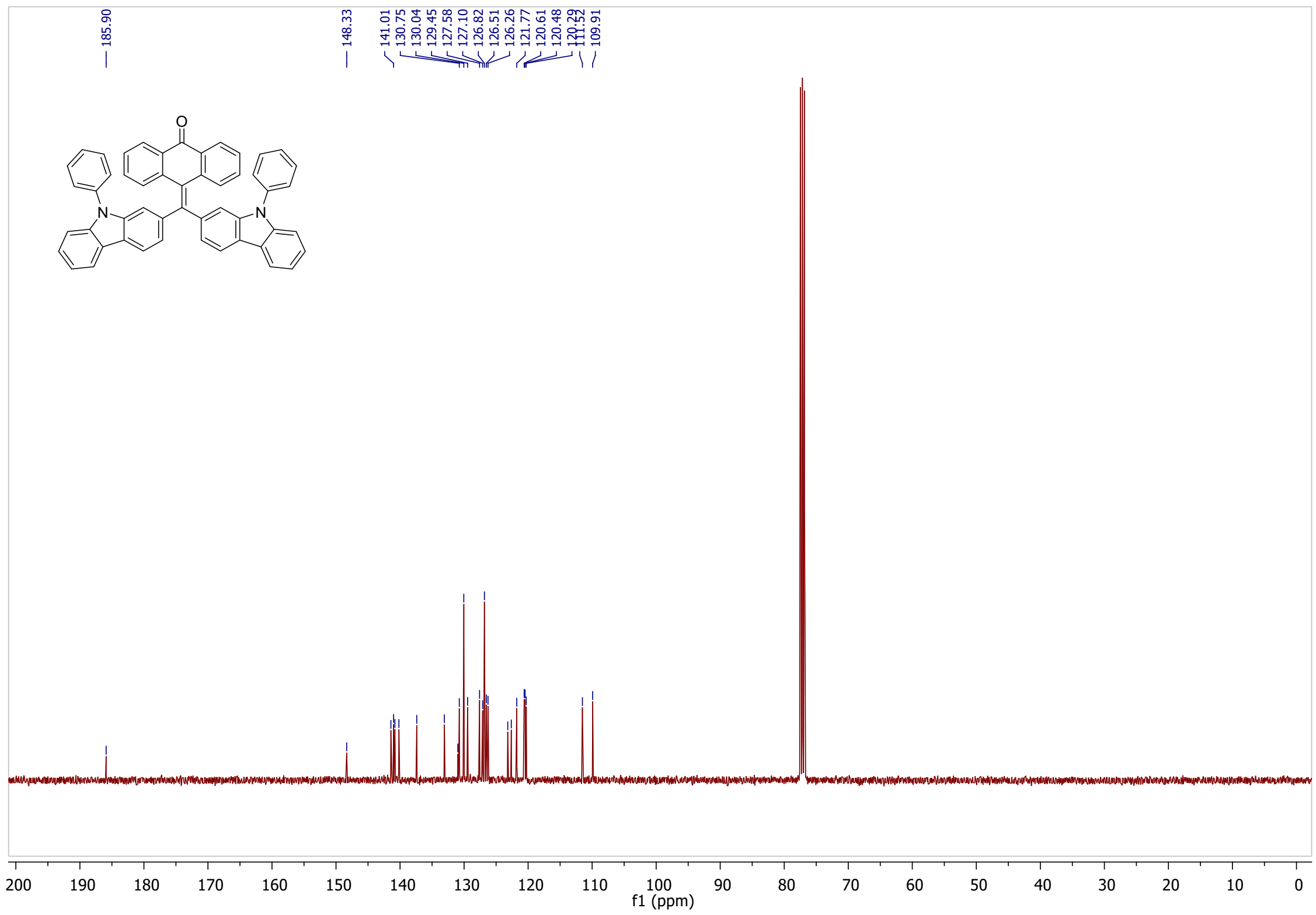

*Figure S6: Compound **3** ^{13}C NMR*

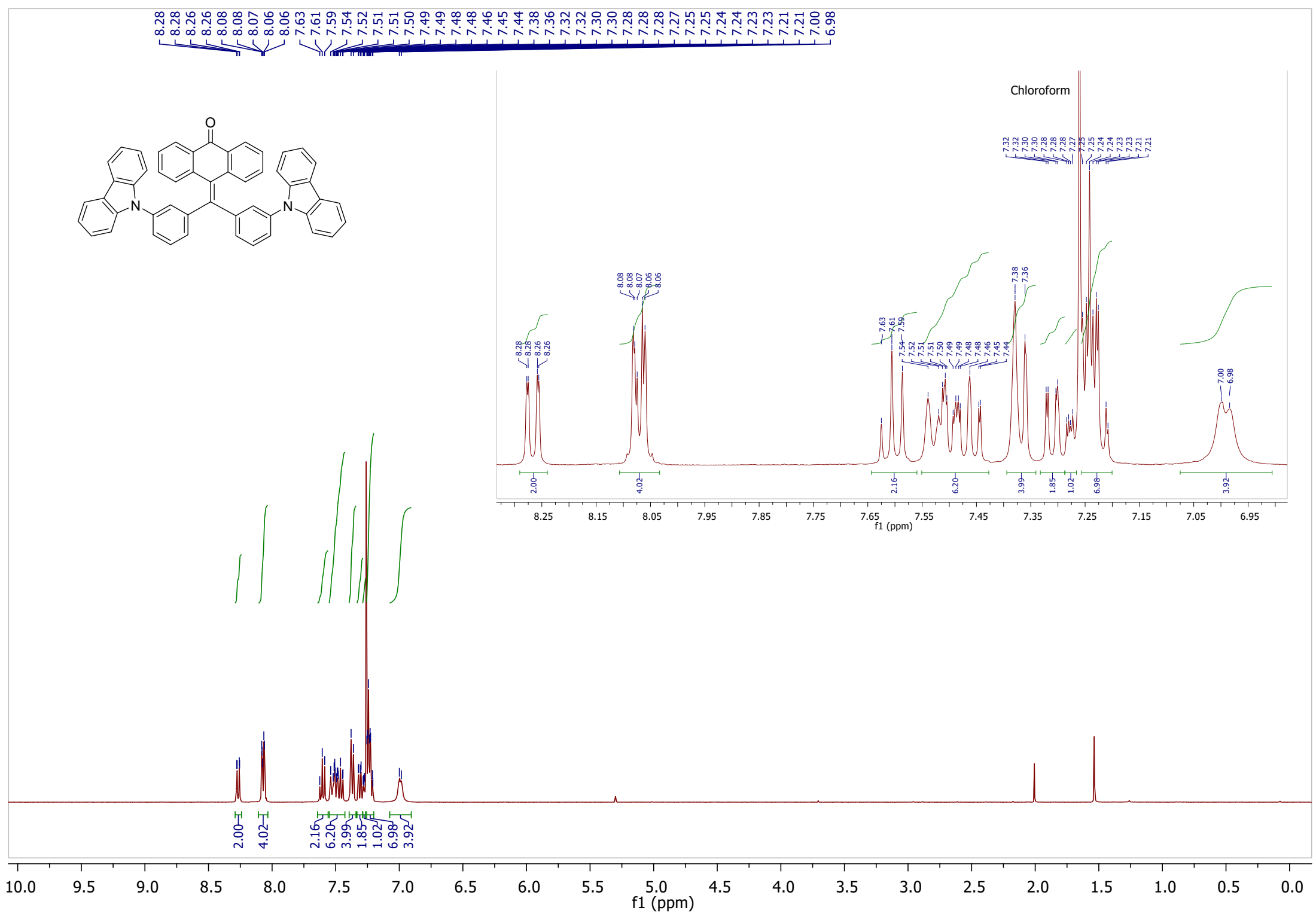

*Figure S7: Compound **4** ^{1}H NMR*

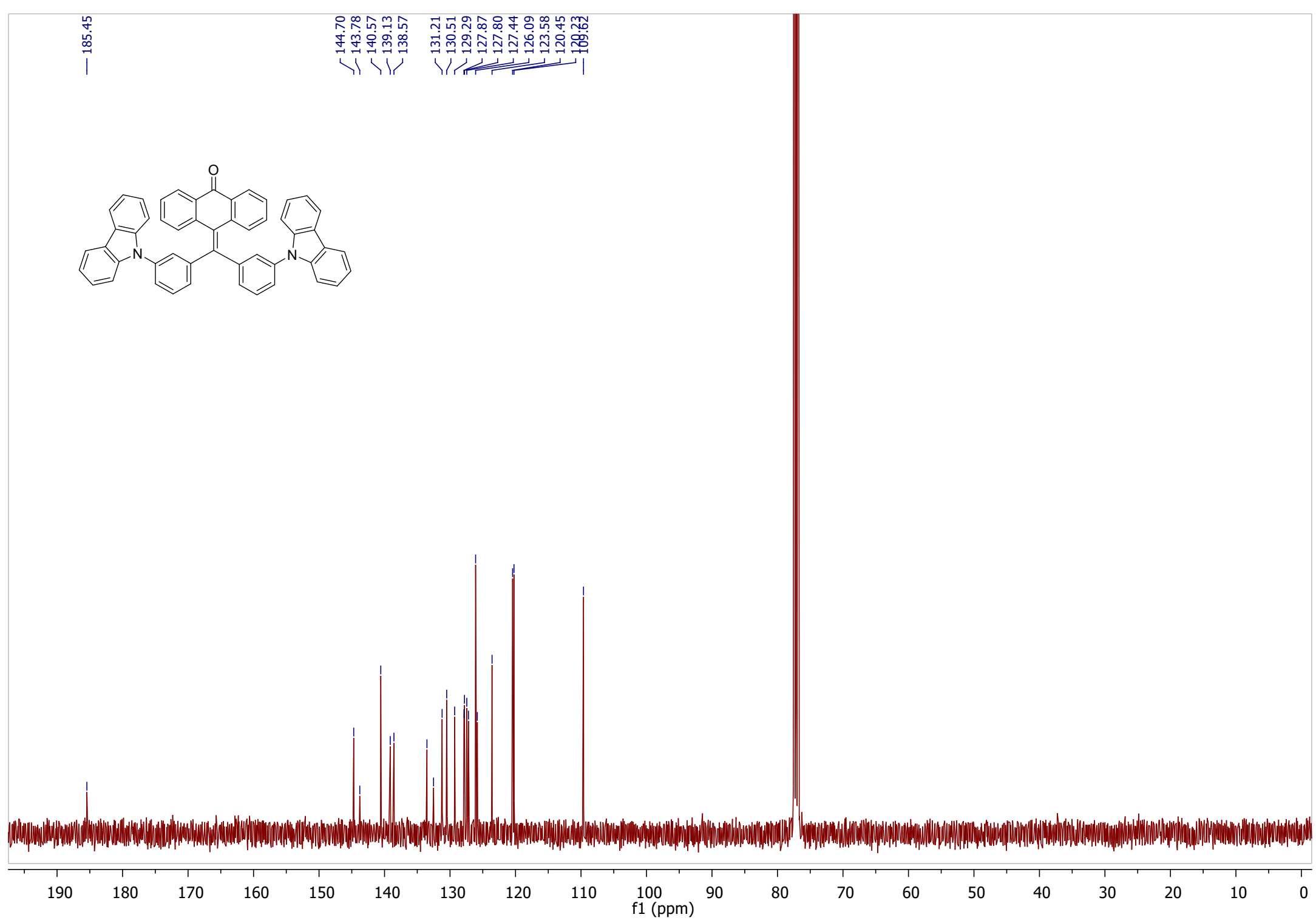

*Figure S8: Compound **4** ^{13}C NMR*

4. Mass Spectra

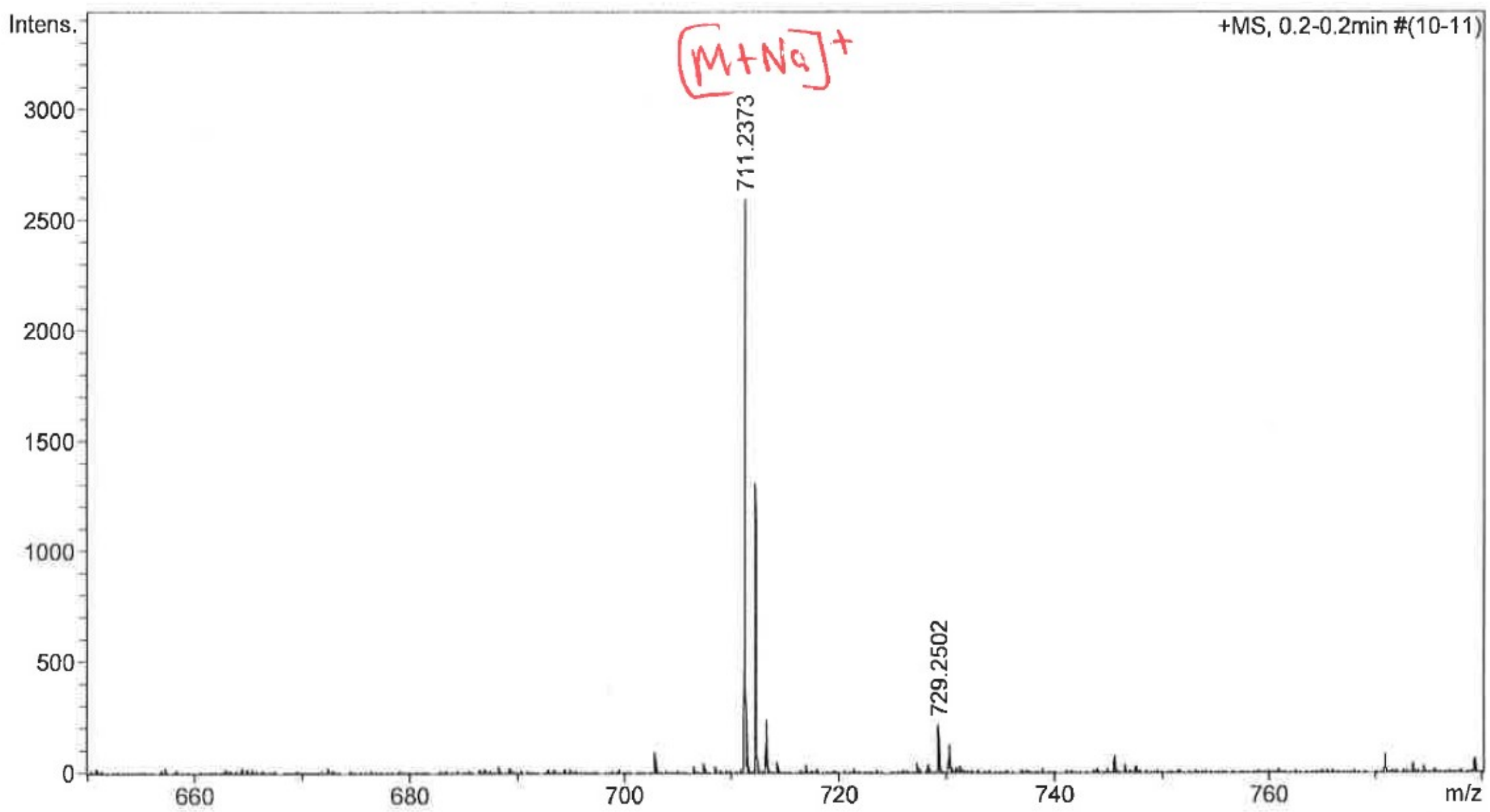

*Figure S9: Compound **1** mass spectrum*

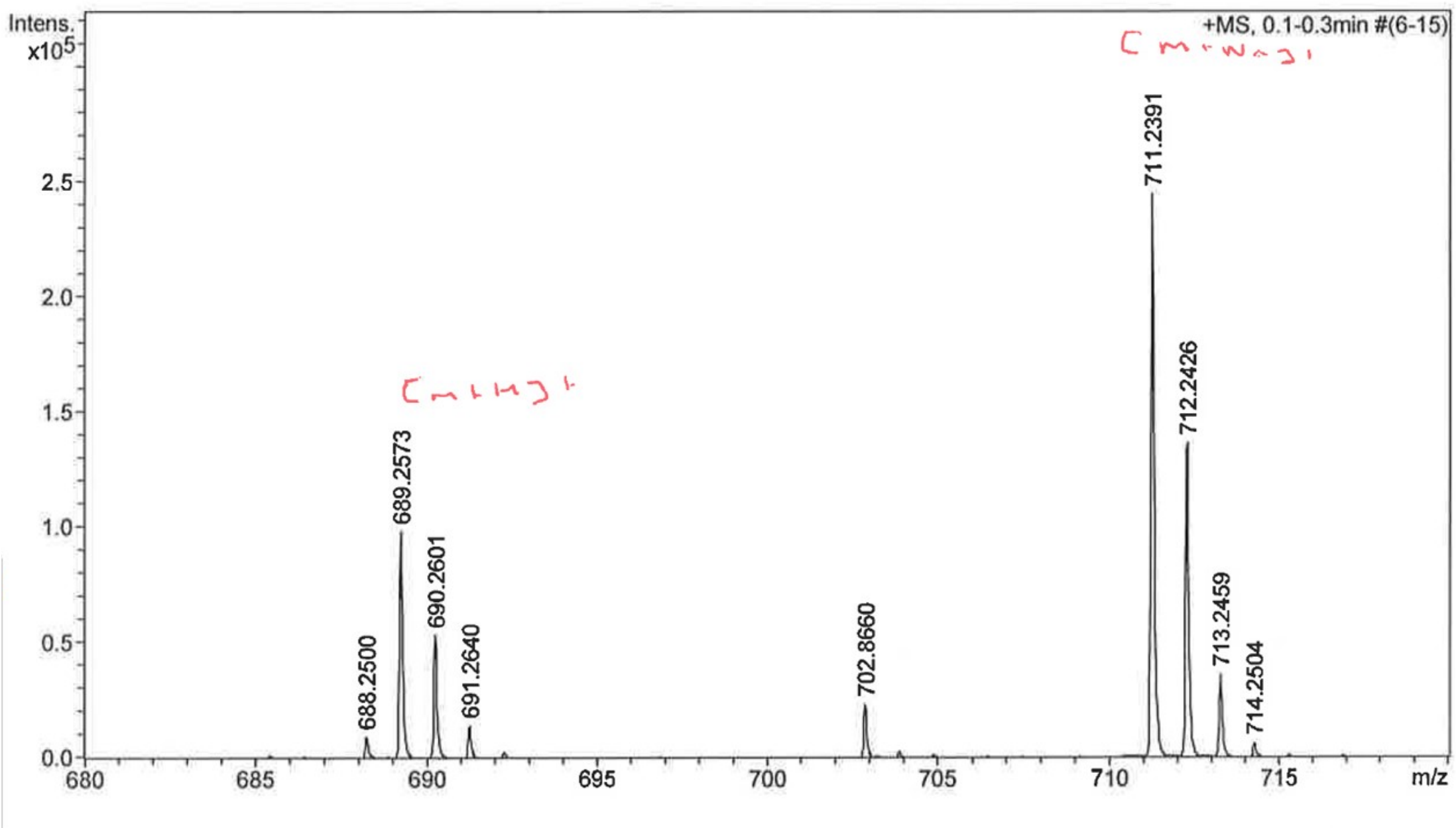

*Figure S10: Compound **2** mass spectrum*

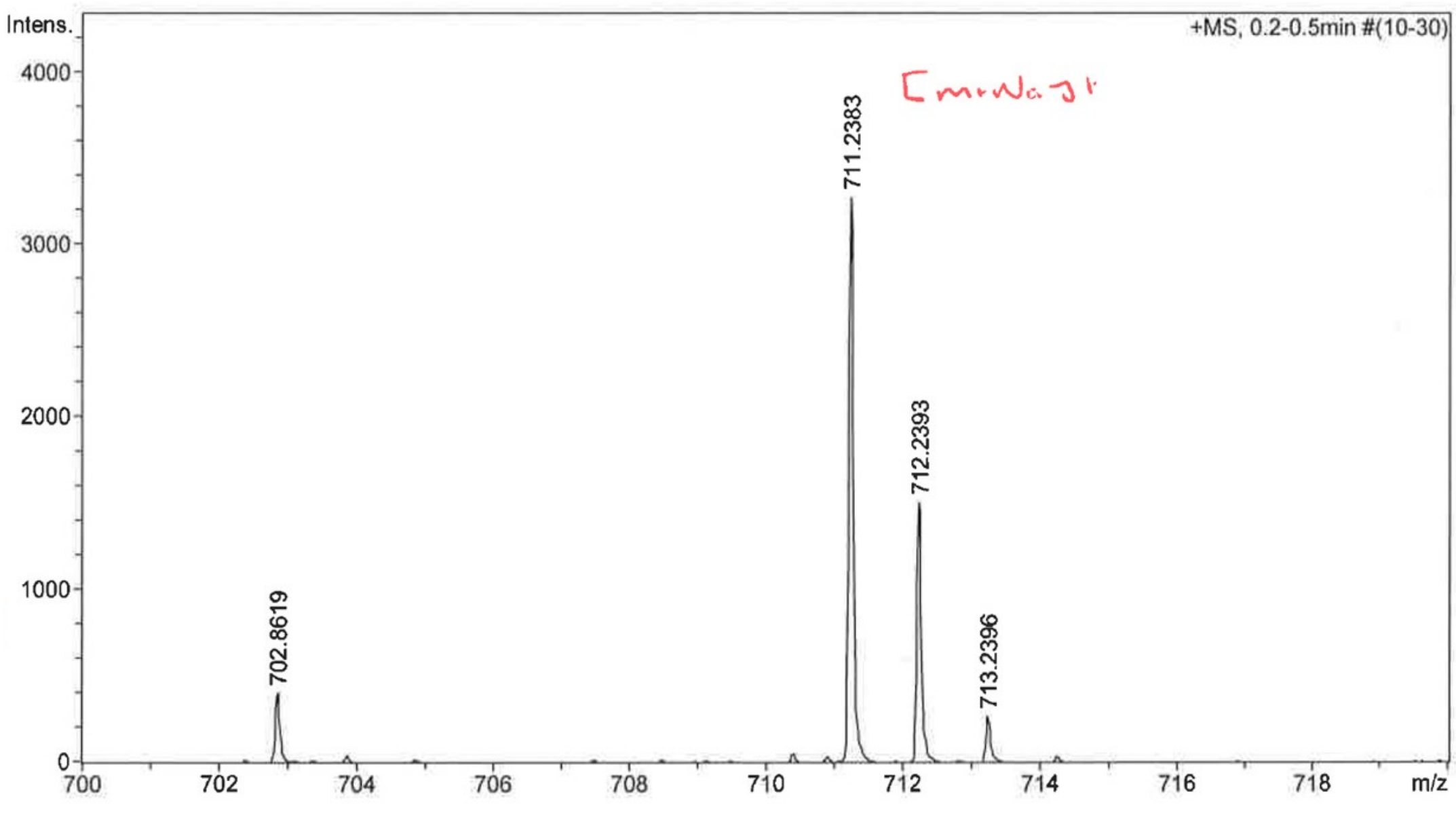

*Figure S11: Compound **3** mass spectrum*

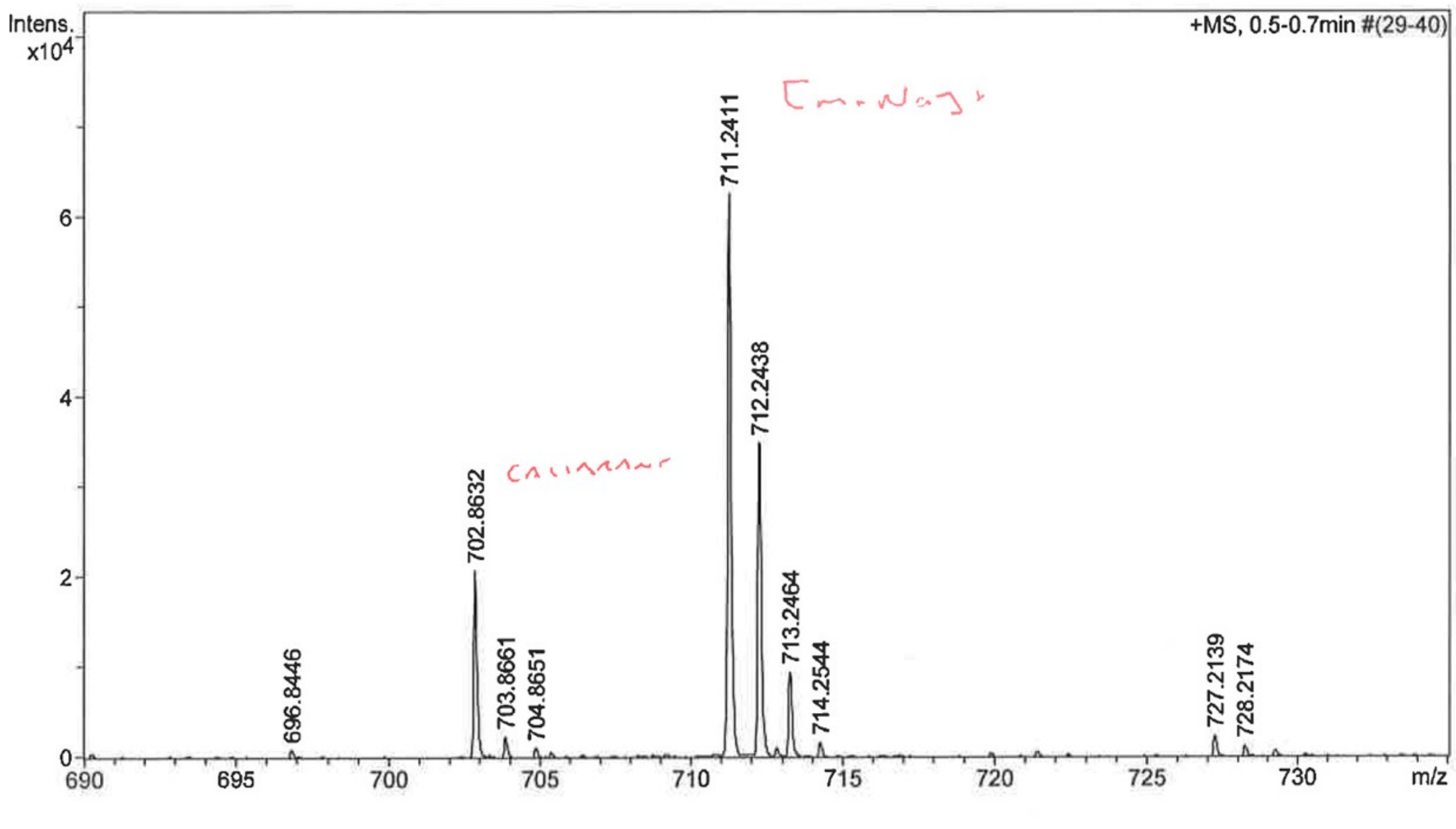

*Figure S12: Compound **4** mass spectrum*

5. Thermal Analysis

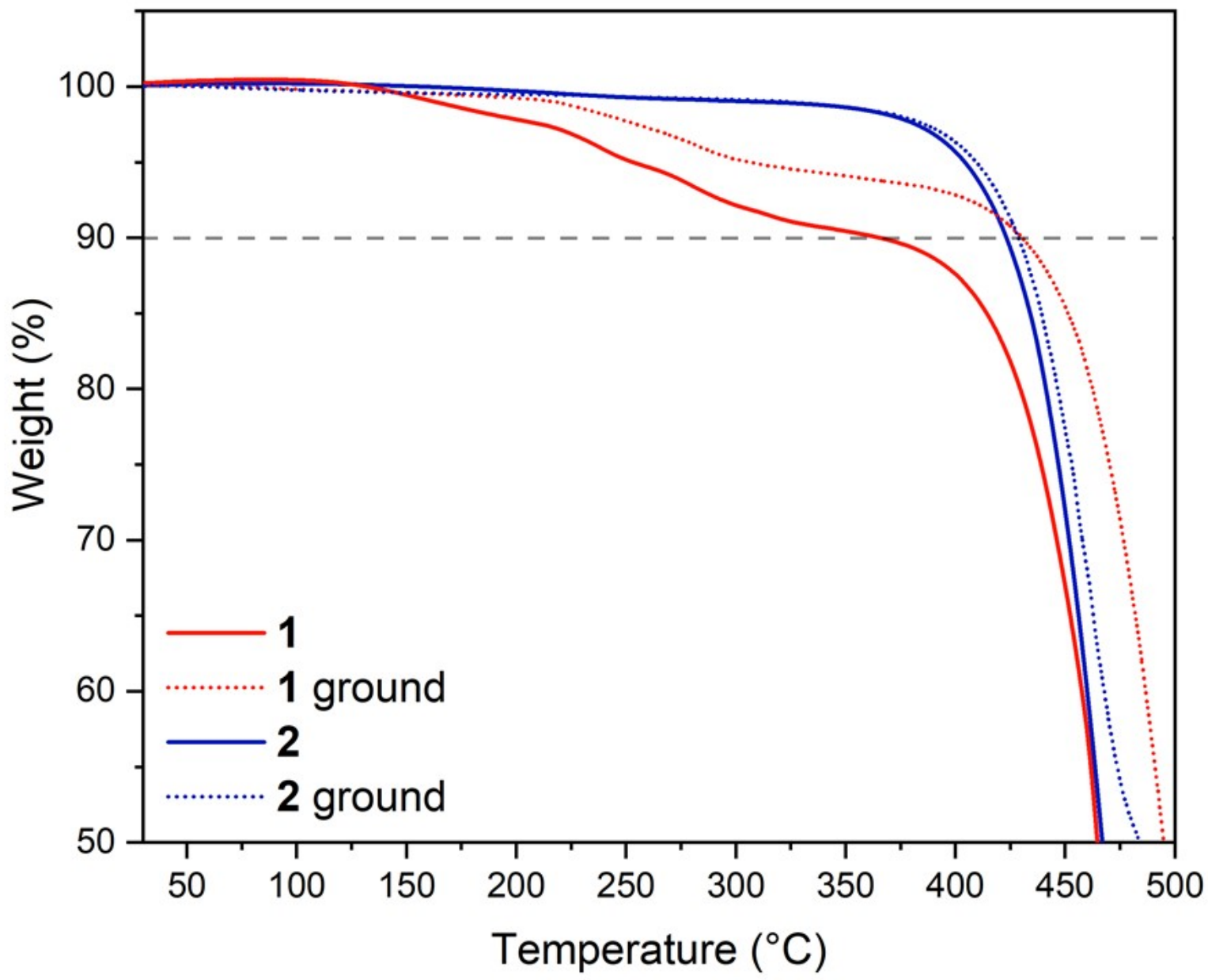

*Figure S13: Thermal gravitational analysis of compounds **1** and **2** before and after grinding*

6. Solvatochroism

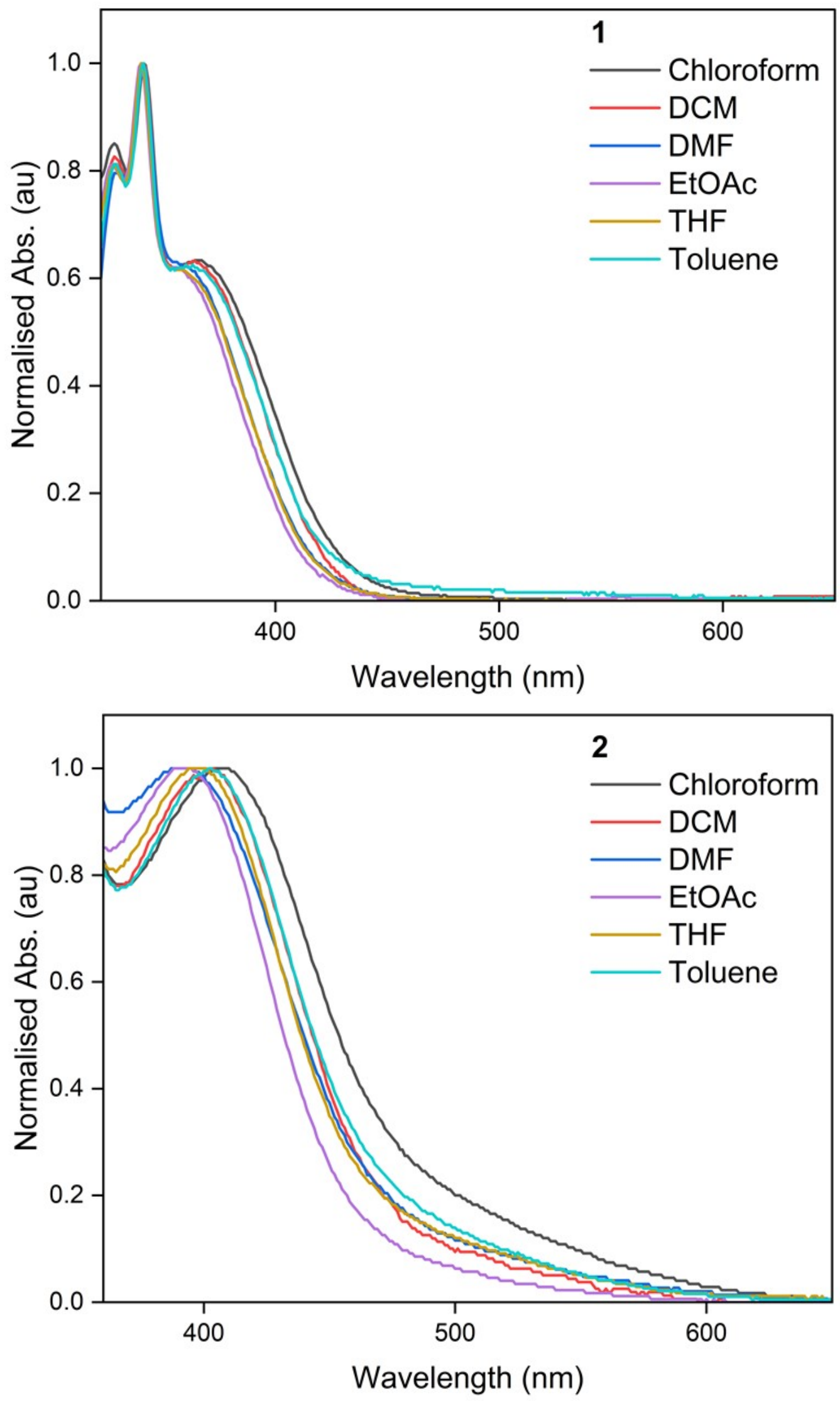

*Figure S14: UV/Vis absorption of compounds **1** and **2** in a range of solvents ($1x10^{-4}molL^{-1}$ solutions)*

7. Computational

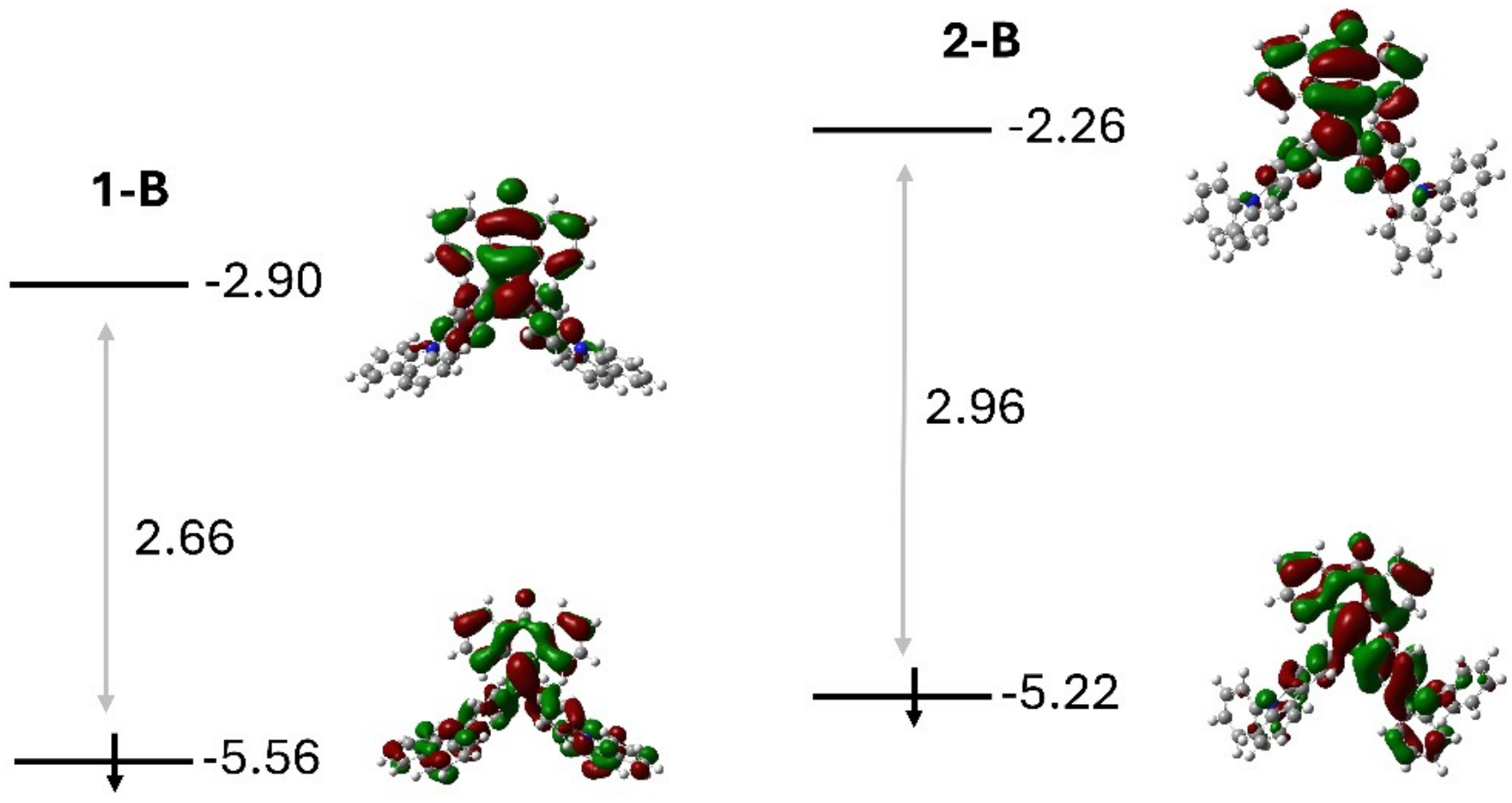

*Figure S15: DFT calculated HOMO and LUMO level for conformers **1-B** and **2-B**.*

8. X-ray Crystal Data

Refinement details

Crystal data, data collection and structure refinement details are summarized for each crystal below.

Computing details

Data collection: *APEX3* Ver. 2016.9-0 (Bruker-AXS, 2016); cell refinement: *SAINT* V8.37A (Bruker-AXS, 2016); data reduction: *APEX3* Ver. 2016.9-0 (Bruker-AXS, 2016); program(s) used to solve structure: XT [3]; program(s) used to refine structure: XL [4]; molecular graphics: Olex2; software used to prepare material for publication: Olex2. [5]

Special details

Geometry - All esds (except the esd in the dihedral angle between two l.s. planes) are estimated using the full covariance matrix. The cell esds are taken into account individually in the estimation of esds in distances, angles and torsion angles; correlations between esds in cell parameters are only used when they are defined by crystal symmetry. An approximate (isotropic) treatment of cell esds is used for estimating esds involving l.s. planes.

Structure report for Compound 2 (CCDC 2553246)

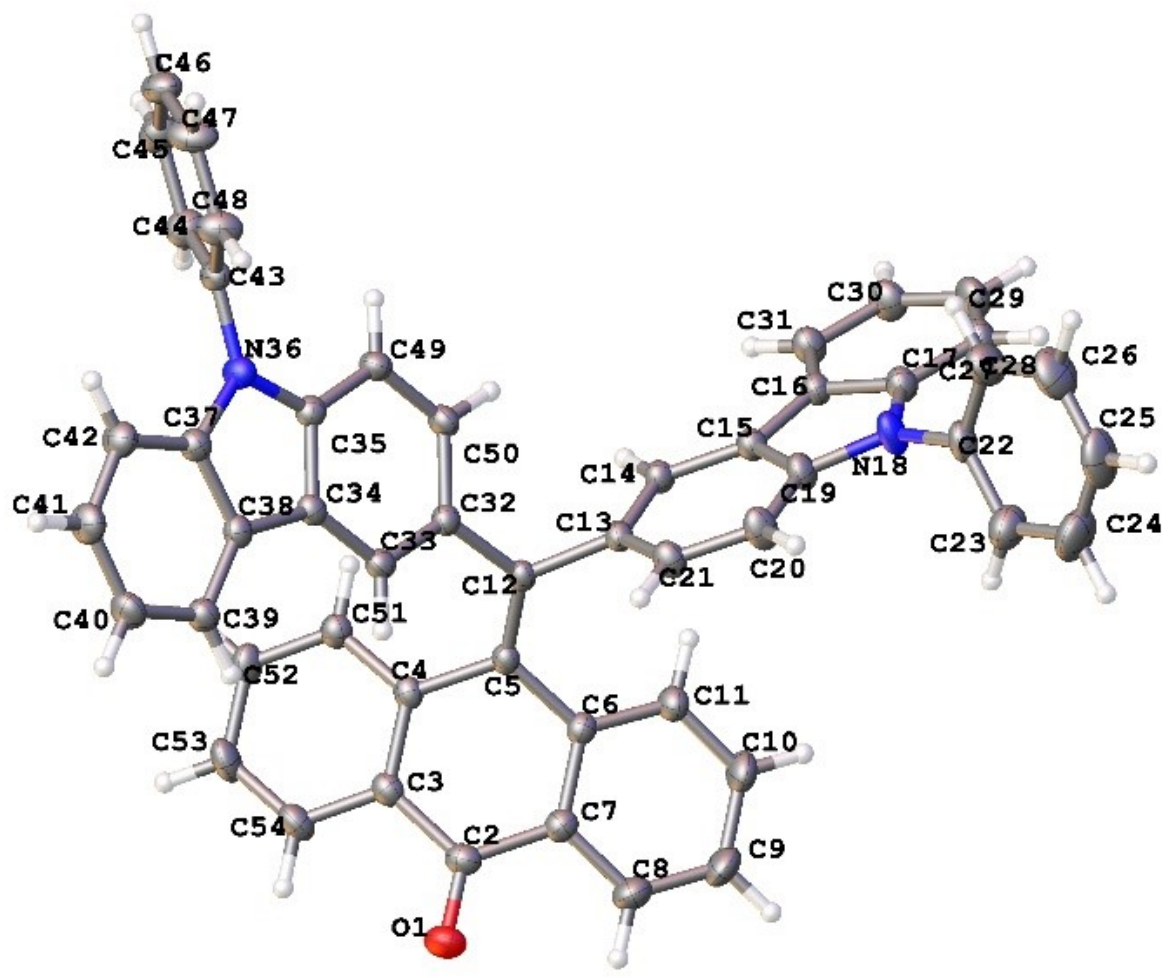

View showing the structure and atom labelling scheme for **2**. Atomic displacement ellipsoids drawn at 50% probability level.

Crystal data

$C_{51}H_{32}N_2O$	$F(000) = 1440$
$M_r = 688.78$	$D_x = 1.271$ Mg m^{-3}
Monoclinic, $P2_1/n$	Mo *K*a radiation, l = 0.71073 Å
a = 9.8168 (2) Å	Cell parameters from 40791 reflections
b = 36.9079 (6) Å	q = 2.1–47.2°
c = 10.4504 (2) Å	m = 0.08 mm^{-1}
b = 108.015 (2)°	*T* = 150 K
V = 3600.74 (12) $Å^3$	Block, orange
Z = 4	0.5 × 0.44 × 0.28 mm

Data collection

XtaLAB Synergy R, HyPix-Arc 150 diffractometer	7940 independent reflections
Radiation source: micro-focus sealed X-ray tube, Mova (Mo) X-ray Source	7267 reflections with *I* > 2s(*I*)
Mirror monochromator	$R_{int} = 0.018$
Detector resolution: 10.0000 pixels mm^{-1}	$q_{max} = 27.1°$, $q_{min} = 2.1°$
w scans	*h* = -12®12
Absorption correction: gaussian *CrysAlis PRO* 1.171.43.90 (Rigaku Oxford Diffraction, 2023) Numerical absorption correction based on gaussian integration over a	*k* = -45®47

multifaceted crystal model Empirical absorption correction using spherical harmonics, implemented in SCALE3 ABSPACK scaling algorithm.	
T_{min} = 0.179, T_{max} = 1.000	l = -13®13
40938 measured reflections	

Refinement

Refinement on F^2	Primary atom site location: dual
Least-squares matrix: full	Hydrogen site location: inferred from neighbouring sites
$R[F^2 > 2s(F^2)]$ = 0.038	H-atom parameters constrained
$wR(F^2)$ = 0.092	$w = 1/[s^2(F_o^2) + (0.0374P)^2 + 1.3789P]$ where $P = (F_o^2 + 2F_c^2)/3$
S = 1.03	$(D/s)_{max}$ = 0.001
7940 reflections	$D\rho_{max}$ = 0.27 e Å^{-3}
487 parameters	$D\rho_{min}$ = -0.18 e Å^{-3}
0 restraints	

Structure report for Compound 3 (CCDC 2553216)

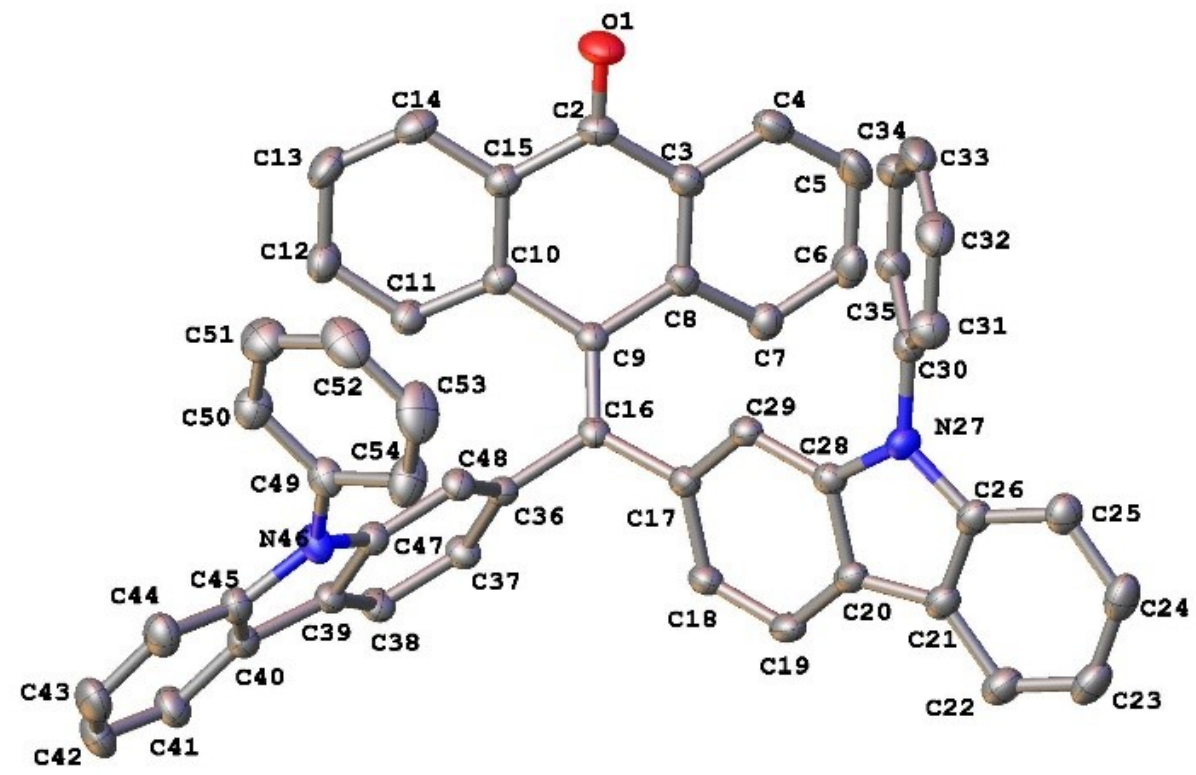

View showing the structure and atom labelling scheme for **3**. Atomic displacement ellipsoids drawn at 50% probability level, hydrogen atoms and lattice hexane molecule omitted for clarity.

Crystal data

$C_{51}H_{32}N_2O{\cdot}0.5(C_6H_{14})$	$F(000)$ = 1540
M_r = 731.87	D_x = 1.254 Mg m^{-3}
Monoclinic, $P2_1/c$	Mo *K*a radiation, λ = 0.71073 Å
a = 14.4537 (3) Å	Cell parameters from 9991 reflections
b = 14.5945 (3) Å	Θ = 2.3–28.2°
c = 19.2586 (4) Å	μ = 0.07 mm^{-1}
b = 107.437 (1)°	*T* = 150 K
V = 3875.81 (14) $Å^3$	Block, yellow
Z = 4	0.24 × 0.2 × 0.17 mm

Data collection

Bruker D8 VENTURE diffractometer	9623 independent reflections
Radiation source: microfocus sealed tube, INCOATEC Iμs 3.0	7540 reflections with *I* > 2s(*I*)
Multilayer mirror optics monochromator	R_{int} = 0.049
Detector resolution: 7.4074 pixels mm^{-1}	$Θ_{max}$ = 28.3°, $Θ_{min}$ = 1.8°
ϕ and ω scans	*h* = -19®19
Absorption correction: multi-scan *SADABS2016*/2 (Bruker,2016/2) was used for absorption correction. wR2(int) was 0.1143 before and 0.0573 after correction. The Ratio of minimum to maximum transmission is 0.8800. The λ/2 correctionfactor is Not present.	*k* = -19®16
T_{min} = 0.656, T_{max} = 0.746	*l* = -21®25

45968 measured reflections	

Refinement

Refinement on F^2	Primary atom site location: dual
Least-squares matrix: full	Hydrogen site location: inferred from neighbouring sites
$R[F^2 > 2\sigma(F^2)] = 0.045$	H-atom parameters constrained
$wR(F^2) = 0.123$	$w = 1/[\sigma^2(F_o^2) + (0.0574P)^2 + 1.008P]$ where $P = (F_o^2 + 2F_c^2)/3$
$S = 1.02$	$(\Delta/\sigma)_{max} = 0.003$
9623 reflections	$\Delta\rangle_{max} = 0.23$ e Å^{-3}
515 parameters	$\Delta\rangle_{min} = -0.21$ e Å^{-3}
0 restraints	

Structure report for Compound 4 (CCDC 2553215)

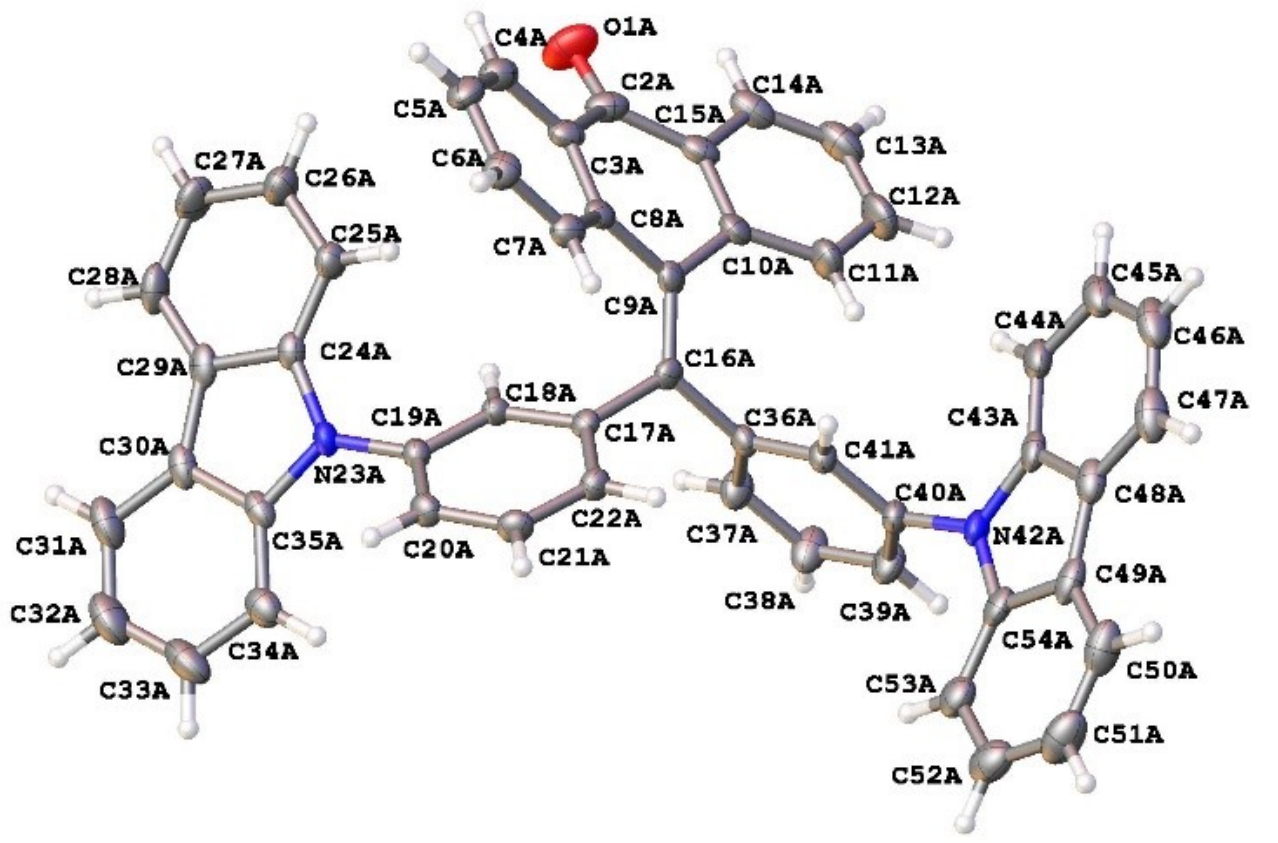

View showing the structure and atom labelling scheme for **4**, atomic displacement ellipsoids drawn at 50% probability level. A second crystallographically independent molecule and 1 molecule of toluene are omitted.

Crystal data

$0.5(C_7H_8)\cdot C_{51}H_{32}N_2O$	*F*(000) = 1540
M_r = 734.85	D_x = 1.257 Mg m^{-3}
Monoclinic, $P2_1$	Mo *K*a radiation, λ= 0.71073 Å
a = 15.9678 (4) Å	Cell parameters from 9920 reflections
b = 10.4389 (2) Å	Θ= 2.5–25.1°
c = 24.2916 (6) Å	μ = 0.07 mm^{-1}
b = 106.462 (1)°	*T* = 150 K
V = 3883.10 (16) Å^3	Column, colourless
Z = 4	0.36 × 0.1 × 0.04 mm

Data collection

Bruker D8 VENTURE diffractometer	12905 independent reflections
Radiation source: microfocus sealed tube, INCOATEC Iμs 3.0	10859 reflections with *I* > 2σ(*I*)
Multilayer mirror optics monochromator	R_{int} = 0.048
Detector resolution: 7.4074 pixels mm^{-1}	Θ_{max} = 25.7°, Θ_{min} = 1.8°
ϕ and ω scans	*h* = -19®19
Absorption correction: multi-scan *SADABS2016*/2 (Bruker,2016/2) was used for absorption correction. wR2(int) was 0.0791 before and 0.0622 after correction. The Ratio of minimum to maximum transmission is 0.9030. The λ/2 correction factor is Not present.	*k* = -12®9

T_{min} = 0.673, T_{max} = 0.745	l = -29®29
33320 measured reflections	

Refinement

Refinement on F^2	Hydrogen site location: inferred from neighbouring sites
Least-squares matrix: full	H-atom parameters constrained
$R[F^2 > 2s(F^2)]$ = 0.050	$w = 1/[\sigma^2(F_o^2) + (0.0145P)^2 + 2.071P]$ where $P = (F_o^2 + 2F_c^2)/3$
$wR(F^2)$ = 0.107	$(\Delta/\sigma)_{max}$ = 0.003
S = 1.03	$\Delta>_{max}$ = 0.35 e Å^{-3}
12905 reflections	$\Delta>_{min}$ = -0.37 e Å^{-3}
1037 parameters	Absolute structure: Flack x determined using 3499 quotients [(I+)-(I-)]/[(I+)+(I-)] (Parsons, Flack and Wagner, Acta Cryst. B69 (2013) 249-259).
1000 restraints	Absolute structure parameter: 0.5 (10)
Primary atom site location: dual	

9. Reference

1. M. J. Frisch, G. W. Trucks, H. B. Schlegel, G. E. Scuseria, M. A. Robb, J. R. Cheeseman, G. Scalmani, V. Barone, G. A. Petersson, H. Nakatsuji, X. Li, M. Caricato, A. V. Marenich, J. Bloino, B. G. Janesko, R. Gomperts, B. Mennucci, H. P. Hratchian, J. V. Ortiz, A. F. Izmaylov, J. L. Sonnenberg, Williams, F. Ding, F. Lipparini, F. Egidi, J. Goings, B. Peng, A. Petrone, T. Henderson, D. Ranasinghe, V. G. Zakrzewski, J. Gao, N. Rega, G. Zheng, W. Liang, M. Hada, M. Ehara, K. Toyota, R. Fukuda, J. Hasegawa, M. Ishida, T. Nakajima, Y. Honda, O. Kitao, H. Nakai, T. Vreven, K. Throssell, J. A. Montgomery Jr., J. E. Peralta, F. Ogliaro, M. J. Bearpark, J. J. Heyd, E. N. Brothers, K. N. Kudin, V. N. Staroverov, T. A. Keith, R. Kobayashi, J. Normand, K. Raghavachari, A. P. Rendell, J. C. Burant, S. S. Iyengar, J. Tomasi, M. Cossi, J. M. Millam, M. Klene, C. Adamo, R. Cammi, J. W. Ochterski, R. L. Martin, K. Morokuma, O. Farkas, J. B. Foresman and D. J. Fox, *Journal*, 2016.
2. T. Nishiuchi, S. Aibara and T. Kubo, *Angewandte Chemie International Edition*, 2018, **57**, 16516-16519.
3. G. Sheldrick, *Acta Crystallographica Section C*, 2015, **71**, 3-8.
4. G. Sheldrick, *Acta Crystallographica Section A*, 2008, **64**, 112-122.
5. O. V. Dolomanov, L. J. Bourhis, R. J. Gildea, J. A. K. Howard and H. Puschmann, *Journal of Applied Crystallography*, 2009, **42**, 339-341.
